# Validation of leaf area index measurement system based on wireless sensor network

**DOI:** 10.1038/s41598-022-08373-z

**Published:** 2022-03-18

**Authors:** Rongjin Yang, Lu Liu, Qiang Liu, Xiuhong Li, Lizeyan Yin, Xuejie Hao, Yushuang Ma, Qiao Song

**Affiliations:** 1 State Key Laboratory of Environmental Criteria and Risk Assessment, Chinese Research Academy of Environmental Sciences, No. 8, Da Yang Fang, An Wai, Chao Yang District, Beijing, 100012 China; 2 State Key Laboratory of Remote Sensing Science, College of Global Change and Earth System Science, Beijing Normal University, No.19, Xinjiekou Wai Street, Haidian District, Beijing, 100875 China; 3 Higher Institute of Computer Modeling and Their Applications, Clermont Auvergne University, Clermont Auvergne, Clermont-Ferrand, France

**Keywords:** Plant sciences, Ecology

## Abstract

Accurate measurement of leaf area index (LAI) is important for agricultural analysis such as the estimation of crop yield, which makes its measurement work important. There are mainly two ways to obtain LAI: ground station measurement and remote sensing satellite monitoring. Recently, reliable progress has been made in long-term automatic LAI observation using wireless sensor network (WSN) technology under certain conditions. We developed and designed an LAI measurement system (LAIS) based on a wireless sensor network to select and improve the appropriate algorithm according to the image collected by the sensor, to get a more realistic leaf area index. The corn LAI was continuously observed from May 30 to July 16, 2015. Research on hardware has been published, this paper focuses on improved system algorithm and data verification. By improving the finite length average algorithm, the data validation results are as follows: (1) The slope of the fitting line between LAIS measurement data and the real value is 0.944, and the root means square error (RMSE) is 0.264 (absolute error ~ 0–0.6), which has high consistency with the real value. (2) The measurement error of LAIS is less than LAI2000, although the result of our measurement method will be higher than the actual value, it is due to the influence of weeds on the ground. (3) LAIS data can be used to support the retrieval of remote sensing products. We find a suitable application situation of our LAIS system data, and get our application value as ground monitoring data by the verification with remote sensing product data, which supports its application and promotion in similar research in the future.

## Introduction

The leaf area index (LAI) is defined as half of all the leaf areas per unit surface area^1^. It can provide quantitative information of structural description for vegetation canopy structure and energy exchange. As an important parameter of vegetation community growth analysis and ecosystem monitoring, LAI has been widely used in agriculture, forestry, ecology, biology, and other fields^2–6^. Therefore, the acquisition of LAI has become essential at home and abroad, especially when researching the above fields. According to the space coverage, we divide the acquisition of LAI into two types: remote sensing satellite image retrieval and ground station measurement.

Satellite sensing provides products over a very large spatial scale, which is a huge advantage to do regional or global research. Song et al. used remotely sensed leaf area index (LAI) data to monitor spatial–temporal changes of winter wheat phenology in response to climate warming across the North China Plain, and they found high spatial heterogeneity of winter wheat phenology in pixel scale across the whole area, which could not be detected in previous site-based studies^5^. Reygadas et al. analyzed long time series of leaf area index data collected by the Moderate Resolution Imaging Spectroradiometer (MODIS) to determine the trend of forest degradation in Mexico^7^.

Although the satellite remote sensing inversion technology of LAI is becoming more and more mature and widely used, it is still indispensable to measure LAI data on the ground. On the one hand, it can provide more detailed and specific vegetation information on the local scale, on the other hand, the ground measurement data of LAI can be used as the validation data for its remote sensing inversion to improve the accuracy of LAI remote sensing products^8,9^.

The LAI ground measurement methods include the direct measurement method and the indirect measurement method^10,11^. The commonly used direct method is the LAI leaf length and width (LAILLW) method, which uses the sampling method to remove leaves from plants, measure single leaf area one by one and finally convert it into leaf area index^12^. When the sample is suitably representative, the results of direct measurements are more accurate, and direct measurements are thus often regarded as the real leaf area index. However, the method inevitably causes damage to plants and it is with the time-consuming and labor-intensive character^13^. Therefore, indirect methods can be introduced. It reduces the damage caused to the vegetation and the environment, and it is also faster than the direct measurement method. According to the measurement, the principle used in the indirect method is usually divided into the radiometric-based method and the image-based method^12^. The LAI2000 and LAI2200 are a series of instruments for analyzing plant canopy parameters and they are common devices for indirect measurement of LAI^14^. Optical sensors are used to measure the transmitted light above and below the canopy, and the leaf area index is automatically derived from a specific calculation model^15^. Although LAI2000 measurement is accurate and quick, it is not compatible with long-term monitoring of the spatial and temporal dynamics of leaf area development^12,16^.

The development and application of wireless sensor network (WSN) technology provide a possibility for automatic continuous observation of vegetation structure parameters. Up to now, many researchers have done studies on the design and product validation of LAI measurement systems based on WSN. Due to the differences in system design and research areas, the conclusions of these verification works are also different. Qu et al. designed an automatic crop structure parameters measurement system^17^ based on WSN in which the parameters were calculated by measuring the canopy transmittance at different solar height angles. However, the data reliability of the system needs to be further tested, because the obtained data is only compared with the LAI2000, rather than the true value acquired via the direct measurement. Guo et al. created an empirical model^18^ that contains the LAI parameter through a large number of measurements in apple orchards, but some determination coefficients are less than 0.9 with low accuracy. Assessment of FAPAR from ESA Sentinel-2 using WSN found considerable discrepancies between image data and measurements especially in forest ecosystems^19^. These models measure fewer parameters and are only compared with the LAI value measured by the indirect method. WSN, as the data for rapid ground monitoring, also has certain reference results for the verification of remote sensing satellite inversion results. Therefore, the model designed in this paper not only collects the leaf area index, but also collects the radiative transfer rate, and uses a more accurate algorithm to calculate the value of LAI.

In addition, the Internet of things has also made some new progress in the field of plant monitoring. Sun^20^ established an intelligent spacing selection model of Internet of things communication nodes under energy-saving constraints. Their hardware model improves the performance of the Internet of things system. Kundu et al.^21^ proposed Deep learning and IoT-based solutions which makes the model more suitable for automating disease detection and proved that the proposed model is effective in providing a low-cost and handy tool for farmers to improve crop yield and product quality. Dhaka et al.^22^ presented a survey of the existing literature in applying deep Convolutional Neural Networks to predict plant diseases from leaf images. They provide a platform for scholars in the field of applying deep learning techniques for the identification and classification of plant leaf diseases.The findings of these studies provide some ideas for our research.

As a result, a set of automatic, continuous, and low-cost leaf area index observation systems (LAIS), which was composed of the hardware structure of the sensor and software part of the image processing have been designed by our team, and the research results have been published in Sensors journal in 2015^23^. Our previous research mainly focused on the sensor layout, data acquisition, transmission, and storage. In data processing, we simply adapted the finite length averaging method^24^ to estimate LAI from images. However, the finite length averaging method is based on the assumption of Poisson leaf distribution, for crop canopy which has a non-Poisson structure and high vegetation coverage^25^, it may cause underestimation of LAI up to 25%^26^. So, as an improvement for the finite length averaging method, our recent research proposed a new formula to estimate LAI from a gap image. The hardware sensor system is also updated with a new technique to acquire the image of better quality. A series of experiments were carried out in Huailai experimental station, Hebei Province. The purpose of this experiment includes not only testing the performance of LAIS equipment and the processing algorithm of the image acquired by LAIS but also validating the remote sensing LAI products based on the ground LAI observation data. Moreover, the work carried out in Huailai experimental station also includes the ground measurement of radiation flux, surface temperature, albedo, and other parameters^27^, as well as the verification of remote sensing products.

This paper mainly focuses on the verification between the data obtained by the upgraded system and the direct measurement methods and satellite remote sensing products, so as to get the applicability of WSN in different situations. The content of this study includes the acquisition of different types of data in the early stage and the accurate calculation of leaf area index–image processing algorithm. Besides, the verification and comparison between different data.By comparing with LAI2000 measurement and remote sensing LAI product, we try to find a suitable situation for the application of our LAIS system and demonstrate its potential for verification of remote sensing product.

## Materials and methods

### Study area

With the advanced observational techniques, abundant data accumulation, and ability to carry on multi-scale experiments, the Huailai Remote Sensing Station and around (for short Huailai Station), located in Huailai, Hebei province, China (40.349°N, 115.785°E), becomes one of the ideal study areas for the observation and validation of the LAI^27^. The Huailai Station is mainly covered by corn and some weeds. So, we mainly use LAIS to monitor the growth cycle of corn (in April 2015, we submitted an application for plant collection permission to Huailai Remote Sensing Station and obtained approval.)

**Figure 1:**
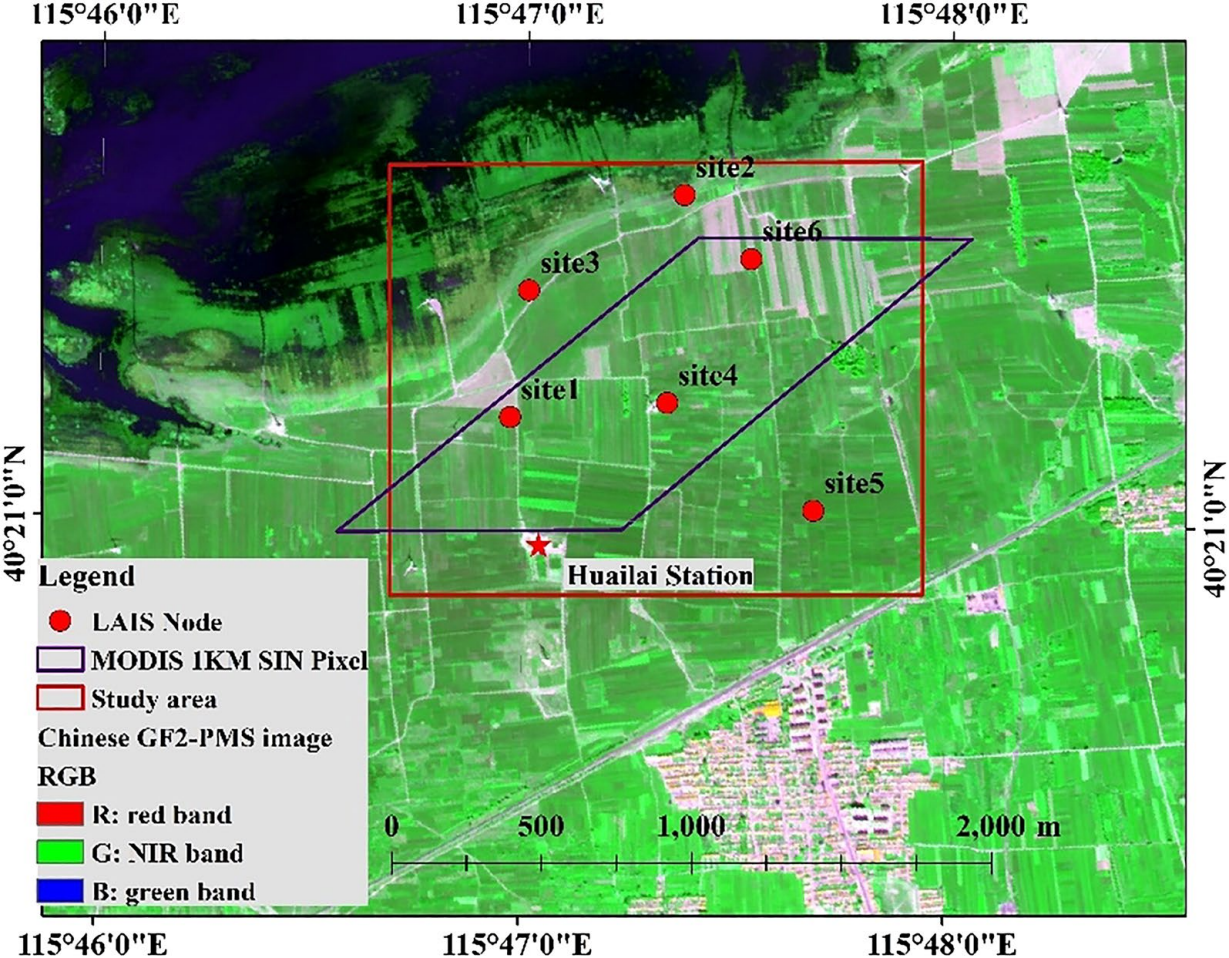
Equipment distribution of WSN vegetation monitoring network in Huailai (red dot represents LAIS Node; purple frame represents the footprint of a MODIS pixel.

Huailai WSN vegetation monitoring system includes 6 sets of monitoring equipment, and its distribution is shown in Fig. 1 as follows, in which red dot represents LAIS Node, purple frame represents MODIS pixel, red frame represents observation area. The observation system is designed for the application of remote sensing pixel scale authenticity tests. The observation scale is a 1 km MODIS pixel on the pixel scale, and the actual coverage area is 2 km * 1.5 km. The six sets of equipment cover the core area of the test station and the surrounding typical growth plot, which is a good representative of the 1 km pixel scale.

**Figure 2:**
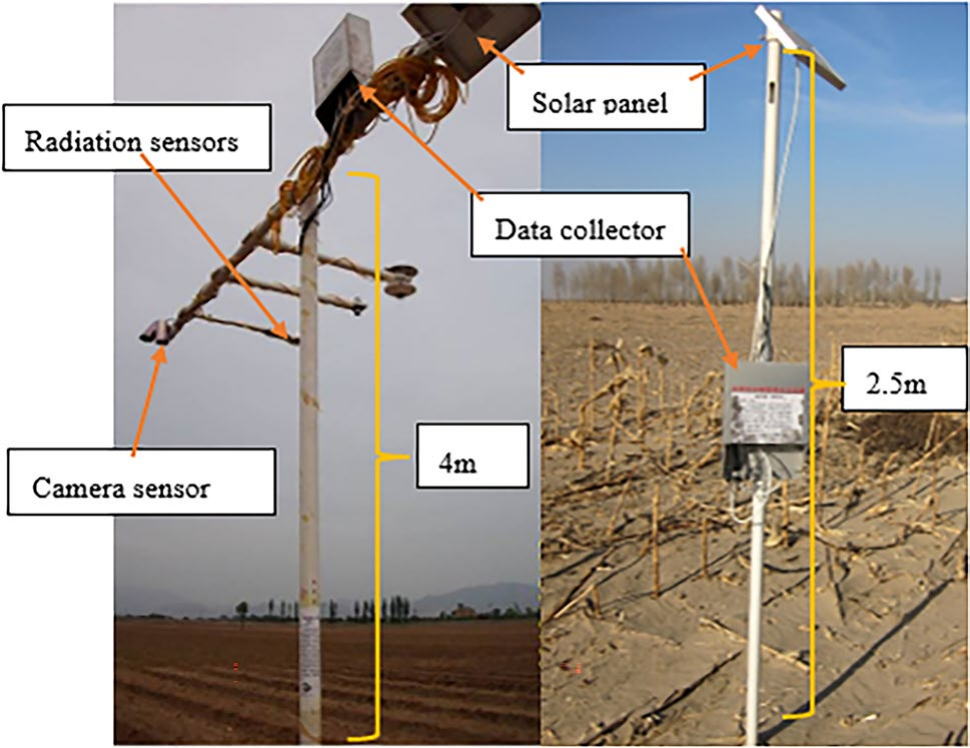
The design of the LAIS node.

Each piece of equipment consists of two cameras which were only one camera with two different angles in previous work^23^ set up at a height of 2.5–4 m above the ground (Fig. 2), one for vertical downward observation and the other for inclined observation, which can take canopy photos regularly every day at its fixed position. The observation system obtained the photos of the corn canopy from May to August, but the corn did not grow in August. Therefore, in this study, we selected the photos taken by the vertical observation camera of the corn sample plot in the experimental station from May to July 2015.

### Related work—data acquisition

#### Data collection using LAIS

**Figure 3:**
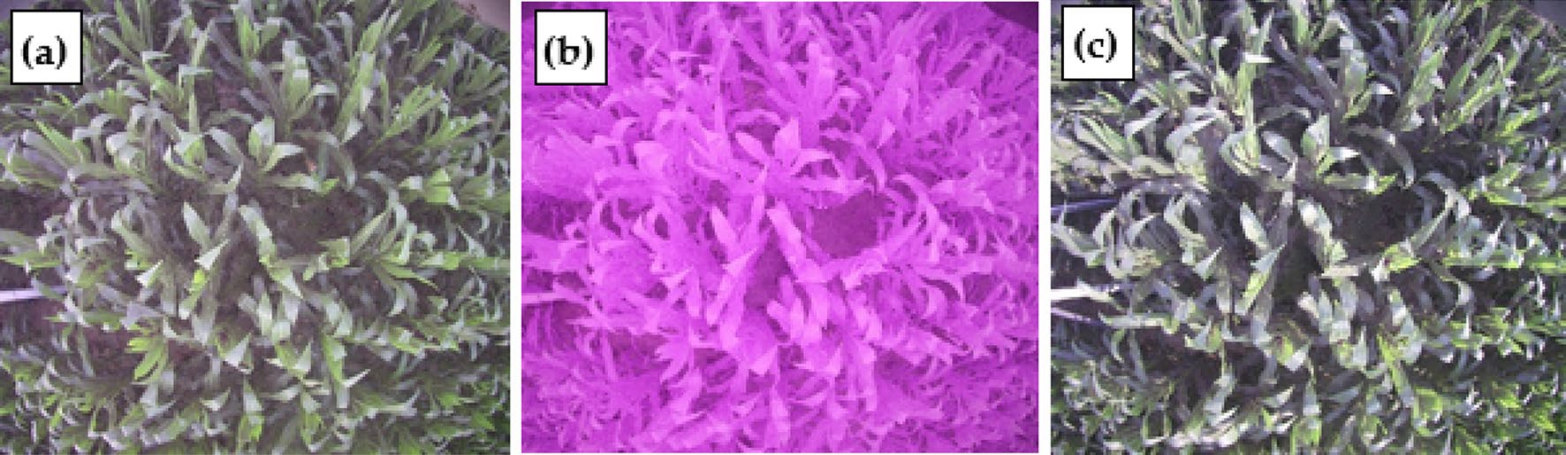
Three images on July 2 of site 1: (**a**) and (**c**) are true-color images obtained at 05:31 a.m. and 6:32 p.m., and (**b**) is a false-color image when the blue filter is removed at 1:28 p.m.

The data collection complies with the plant guidelines statement: "LAI-2000 Plant Canopy Analyzer Instrution Manual" (Supplementary Information 2) (https://www.licor.com/env/, Last visit time: 21 October 2021). Existing facilities such as the high poles and the wireless sensor network in the experimental station have proved convenient for the installation of the LAI measurement system. LAIS uses the GEO001 digital serial camera that is suitable for a variety of embedded image acquisition modes. The specification of the camera includes: the total field of view is 120°, the maximum image size is 2176 * 1920 (approximately 5 million pixels), mounted at a height of 3 m, the spatial resolution at ground level is about 3 mm. The acquired image is simultaneously stored in a flash card in two formats: the JPEG format merits in less file size thus suitable for quick wireless transfer; the RAW format, which is the user data in our analysis, contains 3 channel binary image in 10 bits bit-depth. Compared to our previous work, an important new feature of this camera is the programmable cut-off filter. As we know, unlike scientific sensor which has the precise spectral response to each band, the digital camera is cheap and can only acquire the so-called RGB image. Usual digital cameras have one NIR cut-off filter to exclude the near-infrared light. The GEO001 camera, which was a commercial camera produced by Zhongshan Yunteng Photographic Equipment Co., Ltd, has two cut-off filters: one is the NIR cut-off filter, another is a blue cut-off filter. Switching on the NIR cut-off filter results in an ordinary color image as in a usual household digital camera. While the blue cut-off filter is switched on and NIR cut-off filter is switched off, near-infrared light is allowed to reach the detector array and blue light is blocked, resulting in false-color images as in Fig. 3b. Adding near-infrared light can increase illumination in the shadow area, and blocking blue light can alleviate the disturbance of sun glint, so, switching to a blue cut-off filter helps to improve the image quality when the direct sunlight is strong such as around noon time.

To acquire an image in the best illumination condition and avoid the influence of rain or other unsuitable weather, the image acquisition device based on WSN was set up to acquire images three times per day: 5:30 a.m., 1:30 p.m., and 6:30 p.m. According to our experience, when the canopy is open (sparse vegetation), usually images acquired at 6:30 p.m. are the best for classification because the direct sunlight is weak; when the canopy is closed (dense vegetation), the illumination on the soil background is very poor in all time, and classification is difficult. So, the camera is programmed to switch to a blue cut-off filter when acquiring images at 1:30 p.m., while the images acquired at other times were with NIR cut-off filter, resulting in true color images, as shown in Fig. 3.

#### LAILLW data and LAI2000 data

To evaluate the accuracy of the improved finite length averaging method proposed in this study, a field experiment was carried out to measure LAI by manual sampling (Supplementary Information 3,4). A field sampling scheme covering the corn growing season (late May to early July) was designed (Supplementary Information 1). The LAI of corn in the experimental area was measured by the quadrat harvesting method, and the validation data of LAI of corn in each growth period were obtained. Considering the rapid growth of the corn, the sampling experiment period was set as 1 week, but due to the actual work in summer and the influence of rainfall, six effective measurements were carried out in the field experiment: May 30, June 7, June 13, June 20, July 4 and July 16.

The LAILLW method, which is also known as the shape factor method, involves outdoor and indoor measurements. The formulas are:

1 $${\text{L}} = {\text{S}}*{\text{N}}$$

2 $${\text{f}} = {{\text{S}} /{\left( {\sum\limits_{i = 1}^m {{\text{len}}*{\text{wid}}} } \right)}}$$

where L represents the leaf area index, S refers to the area of a single plant, and N refers to the number of plants in a unit area. The shape factor ƒ is the ratio of the S to the value multiplied by the length and width of all leaves in the plant.

To reduce measurement errors, 10 plants were selected in the sample, and the length and width of each leaf on each corn were recorded with a ruler. To obtain the shape factor, representative corn plants were cut next to the sample (not in the image coverage area) and the true area of each leaf was obtained by software, and the shape factor was derived from this^23^. Through the length and width of 10 strains measured in the field, and the shape factor obtained, the total leaf area of 10 corns can be calculated, and the average leaf area of one plant is finally obtained. The LAI value under the LAILLW method is obtained.

Using the difference between the solar radiation values of the upper and lower canopies, the LAI2000 canopy analyzer can obtain LAI and set up a corresponding point folder to save the measured data for subsequent collation. 10 measurement points were selected for each site, and the average value was the final result for each site. To reduce the effects of the solar altitude angle on measurement accuracy, the experiments were repeated every two hours.

To make it easier to record the date of data acquisition, the data were summarized in the order day of the year (DOY). For example, 30 May 2015 is the 150th day in the year and its DOY is 150. The DOY information of data acquisition using the LAILLW method and LAI2000 is specifically shown in Table 1.

**Table 1:** The DOY information of data acquisition using the LAILLW and LAI2000.

Site	LAILLW	LAI2000
1	150/158/164/171/185/197	150/158/164/171/197
2	150/158/164/171/185/197/213	171/185/197/213
3	150/158/171/185/197/213	171/185/197/213
4	150/158/164/171/185/197	158/164/171/185/197
5	150/158/164/171/185/197/213	158/164/171/185/197/213

#### MODIS LAI data

MODIS leaf area index data was downloaded from the United States Geological Survey (https://modis.gsfc.nasa.gov/data/dataprod/mod15.php), named MCD15A2Hv006. It is an 8-day composite dataset with a 500-m pixel size. The algorithm chooses the best pixel available from all the acquisitions of both MODIS sensors located on NASA’s Terra and Aqua satellites from within the 8 days.

In the comparison of MODIS LAI data, as the pixel of the satellite product is in 500 m resolution, it is not recommended to directly compare single node LAIS measurement with the MODIS LAI product because of the scale mismatch. Though complicated upscaling approaches have been discussed and implemented in Huailai station for other parameters^28^, it is not the purpose of this study So, we simply averaged the LAI in all the LAIS nodes to compare to the average MODIS LAI product in the 3 * 3 nearest pixels (1.5 km * 1.5 km), referred to as MODIS LAI_Mean in a later context, which approximately covers the area of all LAIS nodes. Time matching was carried out by selecting the date of the MODIS product closest to the date of the handheld LAI2000 measurement. The following Table 2 is obtained by taking 3 * 3 pixels closest to the LAIS Nodes.

**Table 2:** MODIS leaf area index of 3 * 3 pixels around Huailai experimental station.

DOY	1	2	3	4	5	6	7	8	9	MODIS LAI_Mean
A2015145	0.3	0.2	0.2	0.2	0.2	0.2	0.2	0.4	0.3	0.244444
A2015153	0.3	0.3	0.2	0.3	0.4	0.3	0.5	0.5	0.6	0.377778
A2015161	0.7	0.8	0.5	0.8	0.8	0.8	0.8	0.8	0.9	0.766667
A2015169	1.1	1.1	1	1.1	1.1	1.1	1.3	1.4	1.1	1.144444
A2015177	1.2	2	1.2	1.8	1.8	2	2	2	1.8	1.755556
A2015185	2.2	3.2	1.8	3.5	2.8	2.8	3.5	3.5	3.4	2.966667
A2015193	1.9	1.9	1.6	2	2	2.2	2.1	2.1	1.7	1.944444
A2015201	3.1	3.4	2.2	4	3.1	3.4	3	3	3	3.133333
A2015209	2.6	2.6	1.7	3	0.8	0.1	3	0.7	0.1	1.622222
A2015217	1.9	1.9	1.6	2	2	2	2.5	2.5	2.5	2.1

### Improved LAIS methods

**Figure 4:**
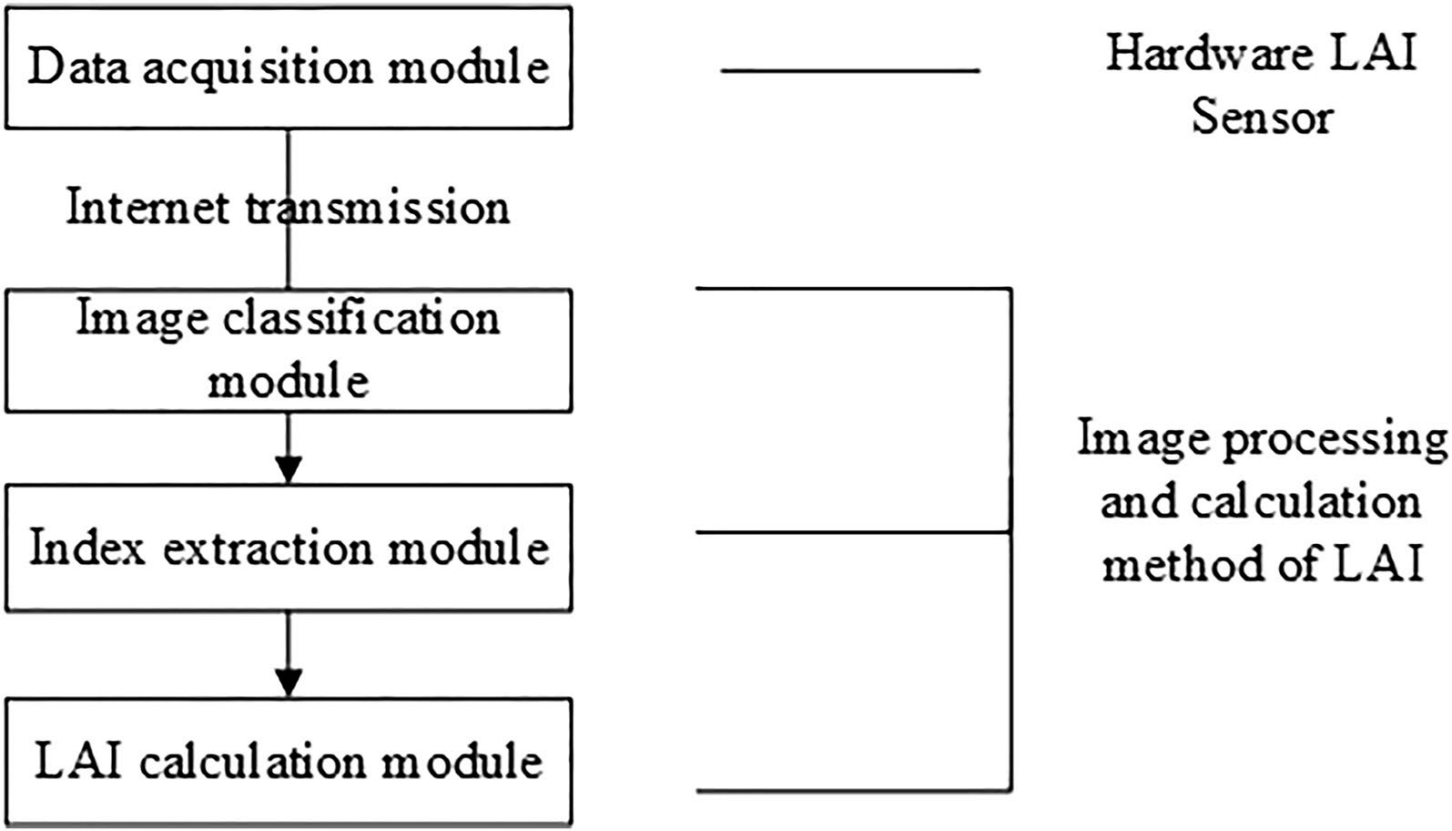
Flow chart of leaf area index measurement system based on WSN.

In previous work, we have deployed sensors and cameras, and also have an automatic image processing and preliminary method of calculating LAI^23^. Figure 4 is a flow chart of our work. The previous articles focused on hardware and system implementation but did not pay much attention to performance. On this basis, we upgrade the image classification method and LAI calculation method, which will be explained in detail below.

#### Image preprocessing and classification methods

Because of weather-related factors such as water vapor and dust or inaccurate exposure, a small number of the photographs are not clear. Besides, some of the image data cannot be decoded because of unstable communications and other factors. Therefore, it is necessary to check and select the photographs that meet the processing requirements before binary image processing. Currently, the selection process is carried out by human visual inspection based on the following principles: (1) when the canopy is open (sparse vegetation), the image at 6:30 p.m. is preferred, when the vegetation the canopy is closed (sparse vegetation), the image at 1:30 p.m. is preferred; (2) if the preferred image is not clear, other clear image acquired on the same day should be used; if all the images are not clear, then this day is marked as a failure.

If we decided to use the image acquired at 1:30 p.m. It is also necessary to convert it from a false-color image to a true-color-like image (as shown in Fig. 3b) in which the leaves are shown in green color. The conversion is carried out by multiplying the vector of DN (digital number) of 3 bands with a coefficient matrix which is provided by the camera manufacturer. Another preprocessing is to choose the near nadir-view area of the image for further processing. As the off-nadir-view area of the image is subject to large geometric distortion as well as saturation of fraction of vegetation cover (FVC), they are not used in this study. The images are clipped to an ROI (region of interest) of about 2 * 2 square meters in ground area, with a maximum view zenith angle less than 30°.

The study of the color spatial distributions of the crop images is helpful for the classification of the images and extraction of the image information. The color of the image pixel is the most direct and effective element that can be used to describe the image^29^. Because the red–green–blue (RGB) color space has the characteristic of a clear and convenient expression of information. When corn leaves are small, the crops in the fields are sparse, and most of them are soil background in the images. The soil in a lower hue is similar to the corn in terms of R and B components, while it has an overlap with the corn in G components when soil is in a higher hue. This makes it difficult to classify sparse corn scenes only by RGB space, so it is necessary to consider the characteristics of hue, luminosity, and saturation (HLS) spatial components.

Statistical analysis showed that the component values of the crop leave in the RGB color space were in the ranges of G > R and G > B while the corresponding values for the soil follow the law that B < G < R^30^. In the HLS color space, the H component has a specific distribution law^31^. Therefore, the distributions of R, G, B, and H components were used as an important criterion for crop image classification^32^.

In this study, two threshold classification methods were proposed, as shown in Table 3 The default thresholds (t1, t2, t3, t4, and t5) were recommended by the system when operating each method.

**Table 3:** The relationship of two threshold classification methods.

Method	Definition
Method 1	(t_1_ < H < t_2_ and S > t_3_) or (G > t_4_) or (R < t_5_and B < t_5_)
Method 2	(G > R + t_1_ and G > B + t_2_) or (G > t_4_) or (R < t_5_ & B < t_5_)

The condition “t1 < H < t2” corresponds to the typical range of H component of green leaves in HLS color space, and “S > t3” is for the occasion when the junction of green leaves and soil in the H component is not very high. The intersection of two conditions can accurately distinguish the general green leaves. “G > t4” can distinguish green leaves with high illumination and “R < t5 and B < t5” can obtain green leaves in the case of dry soil background. The three conditions can get higher classification accuracy. In the experiments, the crop canopy images that were obtained when the leaves are small and the corn is sparse in the field were suitable for use with method 1 and the first four corresponding default thresholds were 80, 160, 18, and 240. When “t5 = − 1” the soil is moist and when “t5 = 40” the soil is a little dry.

When the leaves were larger and they occluded each other, changes in the chlorophyll content and enhancement of the sun-light resulted in a low contrast between the leaf and the background in the true-color images when the growth period reached two months into the panicle stage, so it was no longer appropriate to use classification method 1. The default thresholds in method 2 were 5, 5, − 1, 80, and − 1. False-color images are acquired in raw format. First, need to convert the RAW format to BMP format. Then replace the order of the image bands of G and B in professional software such as ENVI.

**Figure 5:**
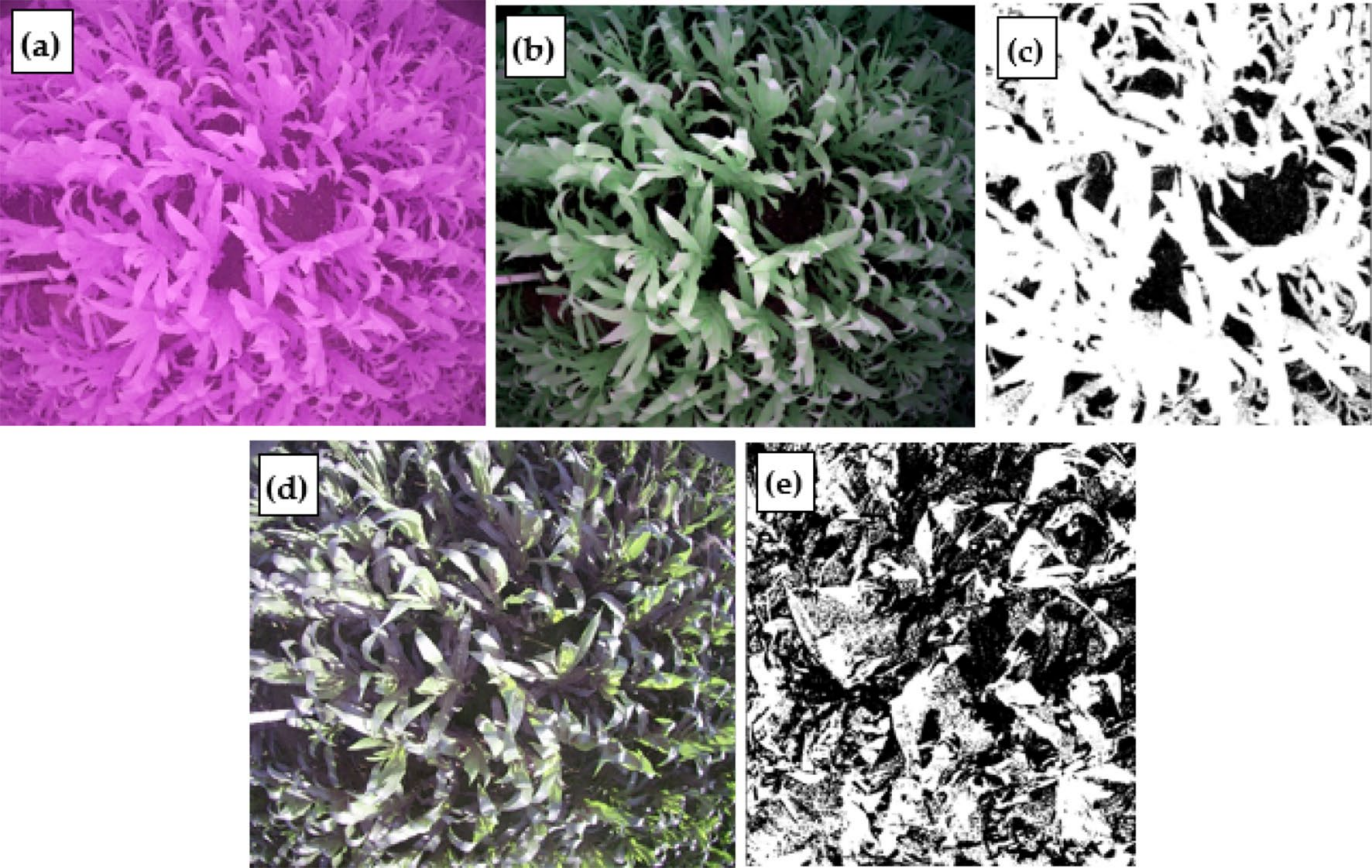
Original images and binary images of site 1 on July 1: (**a**) the original image at 1:30 p.m.; (**b**) preprocessing result of image (**a**); (**c**) the binary image of (**a**); (**d**) original image at 6:30 p.m.; (**e**) binary image of (**d**).

In the classification of crop canopy images, only the sub-region in the center of the photograph was used because of the geometric distortion of the camera photographs, and the corresponding area in each binary image had dimensions of approximately 2 × 2 m. Figure 5 shows the original and binary images of site 1 at 1:30 p.m. and 6:30 p.m. on July 1. By visually comparing the original image with the binary image (Fig. 5), it can be seen that the classification result of the false-color image is better when the leaves are large and occluded, the contrast between the lower leaves and the soil is low. This is also another significant finding in this validation experiment and it’s of practical significance for the application of the proposed system to farmland monitoring.

**Figure 6:**
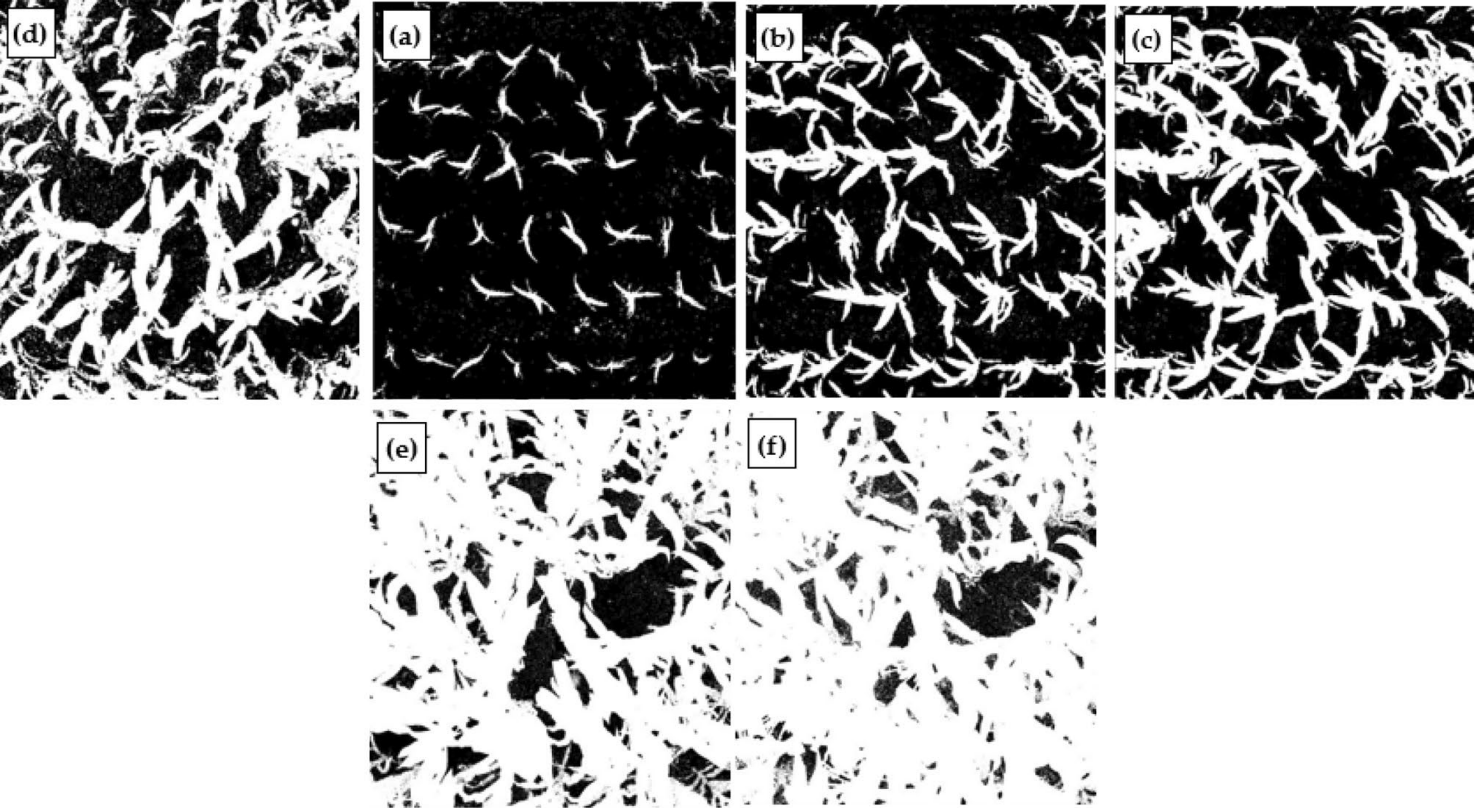
The time series of extracted leaf-cover from the digital images in site 1: (**a**) May 30; (**b**) June 7; (**c**) June 13; (**d**) July 4; (**e**) July 16; (**f**) August 1.

During the application, it is necessary to select the appropriate image as well as classification methods by the practical situation such as the crop type, the crop growth stage, and the image quality. We have mentioned in “Data collection using LAIS” section that when the canopy is sparse, we prefer to use the image at around 6:30 p.m.; if the illumination on soil is too poor as the result of the dense canopy, we prefer to use the false-color image at around 1:30 p.m., and the above color space transformation is required. Sometimes the weather is bad and the preferred images are not of good quality, we need to choose one of the three images at 5:30 am 1:30 p.m., and 6:30 p.m. according to the situation. Currently, this choice needs human visual inspection, but it is a quick and simple operation, and won't waste much time as in the field measurement. Although most of the image processing is automated, human supervision is still essential, as the image quality is important to distinguish leaves from soil background, which is a prerequisite for calculating LAI. Figure 6 shows the binary images for the different dates in site 1.

#### The improved finite length averaging method for LAI estimation

The classification discriminates pixels of green leaf from soil background in the LAIS acquired image. So, the fractional vegetation coverage (FVC) and gap probability, which equals 1-FVC, can be derived by dividing the number of leaf pixels by the total number of pixels in the ROI, assuming the change of view zenith angle within the ROI can be neglected. Theoretically, LAI can be related to FVC through the widely used Beer-Lambert law.

However, the original Beer-Lambert law only applies to uniformly distributed and infinitesimal leaves. For the real canopy, leaf angle distribution and clumping index (CI) should be considered^1,24^. The finite length averaging method was proposed in 1986 to simultaneously estimate LAI and CI from field measurement of canopy gaps. It also applies to gap data generated from canopy photography and still is the recommended method to estimate LAI and CI for crop canopy up to now. The formula can be summarized as:

3 $$L = - \frac{\cos (\theta )}{{mG(\theta )}}\sum\limits_{i = 1}^{m} {\ln \left( {P_{i} (\theta )} \right)}$$

4 $$\Omega = \frac{{m\ln \left( {\frac{1}{m}\sum\nolimits_{i = 1}^{m} {P_{i} (\theta )} } \right)}}{{\sum\nolimits_{i = 1}^{m} {\ln \left( {P_{i} (\theta )} \right)} }}$$

where L denotes LAI and $\Omega$ denotes CI; ${P}_{i}(\theta )$ is the gap probability when observation zenith angle is $\theta$, which is 0 in this case; $G(\theta )$ is related to leaf angle distribution and is normalized projection area of leaf in the observation direction; the footnote $i$ denotes the ${i}{th}$ sample line/sample rectangle, and ${\text{m}}$ is the total number of sample lines/sample squares. When dealing with gap data generated from canopy photography, usually the sample square is adopted instead of the sample line.

However, Lang and Xiang^24^ also pointed out two limitations with this approach: (1) If the gap probability is equal to 0, then the estimation is meaningless as infinity occurs. This is often the case of a high LAI. (2) the method assumes that the leaf size should be sufficiently small relative to the side of the sample square. If the sample square is too small, then the precondition cannot be satisfied, and usually, an overestimation of LAI occurs.

As the camera of LAIS is fixed on the top of a pole, the acquired sample image is of limited area. Then we face the problem of either using a small sample rectangle or the total number of sample rectangles is insufficient. To solve the problem of a small sample square, we proposed empirical formula to replace the log function in characterizing the relationship between gap probability and LAI in the sample square based on computer simulations^33^. The new formulas correct the shortcomings of over-estimation and instability of log function when the canopy is dense and the side length of the sample square is short. The revised formula is:

5 $$\mathrm{L}=\frac{1}{m}\sum_{i=1}^{m}\frac{cos{\theta }_{i}}{G\left({\theta }_{i}\right)}f({P}_{i}\left({\theta }_{i}\right),D)$$

where D denotes the equivalent leaf length, which is defined as the square root of the average area of the single leaf; and $f$ is the proposed empirical formula, in the form of:

6 $$f\left(P,D\right)=\left(1-P\right){P}^{1/{A}_{1}}+\frac{\left(1-{P}^{1/{A}_{2}}\right){(1-P)}^{1/{A}_{3}}}{1/{A}_{4}+{A}_{5}{P}^{{A}_{6}}}$$

7 $${\mathrm{A}}_{i}={a}_{i1}+{a}_{i2}(W/D)+{a}_{i3}\mathrm{log}(W/D) i=\mathrm{1,2}\dots ,6$$

where ${a}_{ij}$ ($\mathrm{i}=1,...,6; j=\mathrm{1,2},3$) are empirical coefficients with values in Table 4. W is the side length of the sample squire. Our study also found that the optimal setting for the side length of the sample square is about 3 times of equivalent leaf length in most cases of crop or grass scenes.

**Table 4:** The coefficients in $f\left(P,D\right)$ estimation formula for fusiform leaf, using sample square.

$i$	$a_{i1}$	$a_{i2}$	$a_{i3}$
1	19.187855	1.156578	− 11.530798
2	0.000651	0.027966	− 0.000930
3	0.389277	0.013358	0.030359
4	4.821348	− 0.046318	0.778747
5	1.215673	0.025448	− 0.030386
6	0.483192	− 0.008525	0.111023

## Results

### Verification and comparison of LAIS data and ground monitoring data

**Figure 7:**
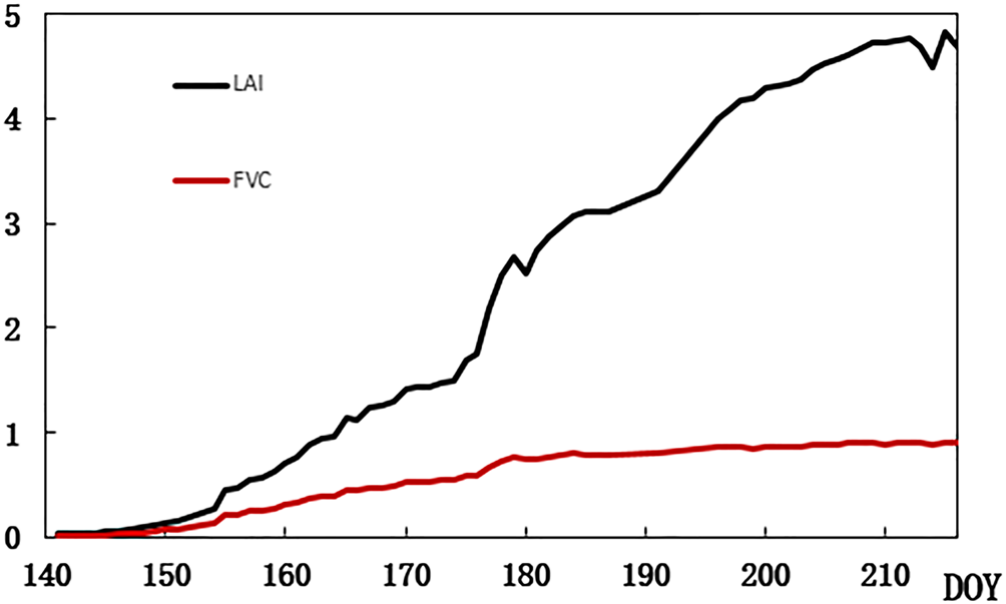
Change curve of LAI and FVC in site 1.

The LAI that was extracted using the improved algorithm and the fractional vegetation coverage (FVC) are shown in Fig. 7. The FVC refers to the percentage of the vertical projection area of vegetation in the total sample area. After one and a half months of corn planting, LAI growth accelerated (see Fig. 7) which is by the typical growth cycle of corn. The WSN-based system increases the time–frequency of data acquisition and detailed analysis of the growth of the crop can be achieved. A small fluctuation was observed in the LAI curve, which was a normal phenomenon. Excessive illumination and a lack of water in the leaves are the main causes of this phenomenon. Both FVC and LAI are unitless ratio values, so are plotted in one picture (Fig. 7).

**Figure 8:**
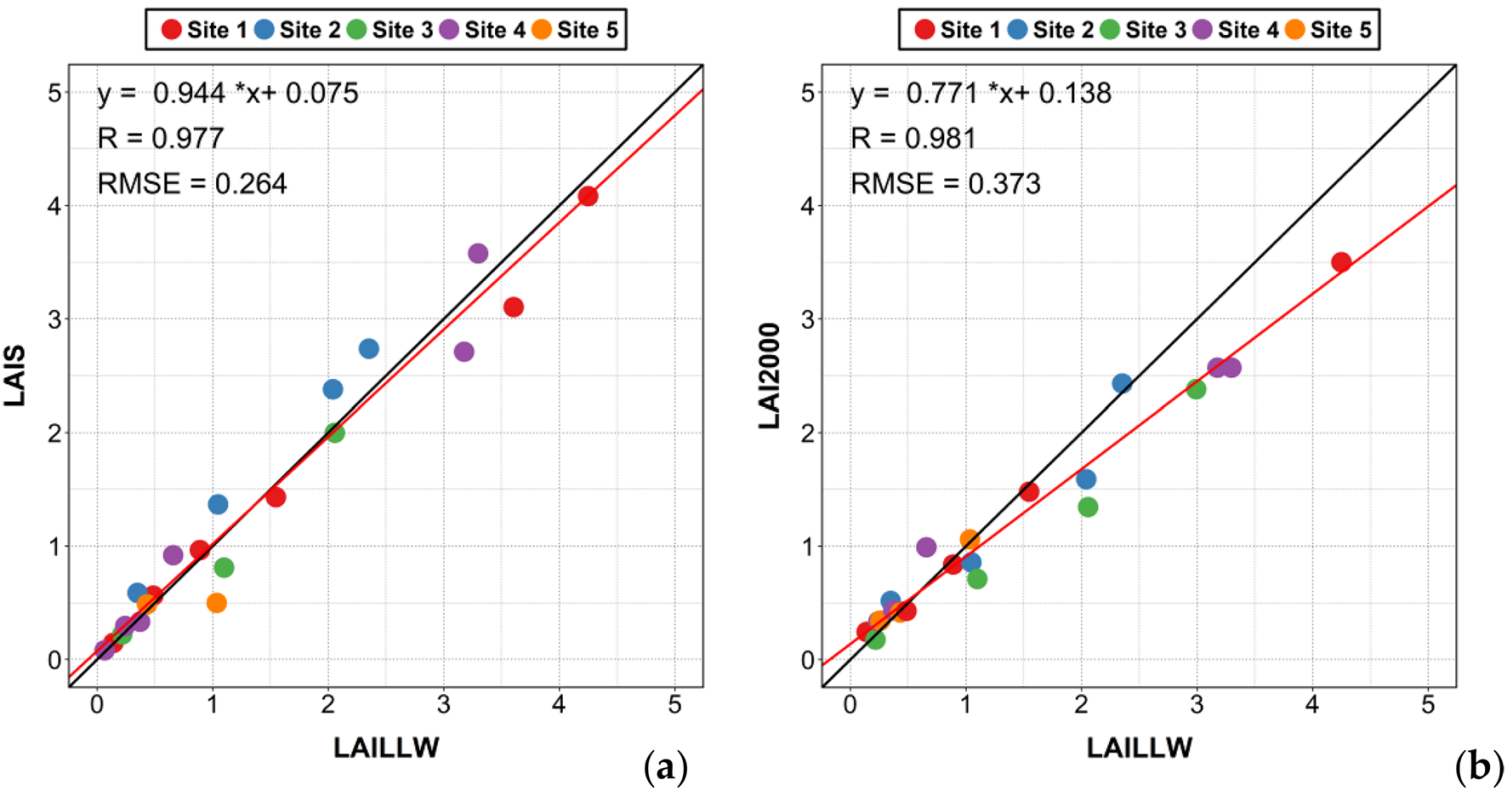
Scatter distribution of the LAI estimation: (**a**) the LAIS; (**b**) the LAI2000.

The LAI data of the area under study can be acquired using a combination of images classification and the improved algorithm. The data obtained by the LAILLW method were considered as true values. A comparison of values obtained using the LAIS, the LAI 2000, and the LAILLW methods is shown in Fig. 8. The results showed that the LAIS data and the LAI2000 data were both in good agreement with the true values using the LAILLW method. The slope of the fitting line of the LAIS is closer to 1 (slope = 0.944), with a very small offset, and the root-mean-square error (RMSE) is smaller (RMSE = 0.264) than the LAI2000. When the LAI is large, there is a significant underestimation of the LAI2000 measurements (see Fig. 8b).

Table 5 shows the acquired LAI data using the LAILLW method and the absolute errors between the other two methods (LAI2000 method and the LAIS method) and the true value. As can be seen from the table, in the early stage of corn growth, the LAIS measurement error is smaller than the LAI2000. The LAI2000 is to calculate the LAI based on the difference in radiance values above and below the canopy, and the LAI2000 is not very sensitive to radiation measurements when the corn leaves are small. Therefore, the measurement of LAI2000 in the early stage of corn growth is not very accurate, and the LAIS method is to obtain LAI by image processing, which can avoid such defects. The main problem of the LAIS method at this stage is that the measured value is higher than the true value. The main reason for this phenomenon is that there are weeds in the acquired image, so the area of the extracted crop is overestimated, and the estimated LAI value is larger than the true value. This factor is not considered when using the LAILLW method for field measurements. When the LAI value is greater than 1, there is a significant underestimation of the LAI2000 measurement, while the LAIS estimate is relatively stable.

**Table 5:** The LAILLW data and the absolute errors between LAI2000 and LAIS data.

LAI_LAILLW	Error_LAI2000	Error_LAIS	DOY
0.1405	+ 0.1305	+ 0.0047	150
0.2425	+ 0.0945	+ 0.0526	158
0.4864	− 0.0574	+ 0.0755	158
0.3727	+ 0.0603	− 0.0392	164
0.4137	− 0.0167	+ 0.055	164
0.8898	− 0.0518	+ 0.0742	164
0.2175	− 0.0415	+ 0.003	171
0.3496	+ 0.1664	+ 0.2372	171
0.6577	+ 0.3323	+ 0.2622	171
1.0343	+ 0.0257	− 0.5361	171
1.047	− 0.19	+ 0.3205	171
1.5469	− 0.0669	− 0.115	171
1.0983	− 0.3883	− 0.2882	185
3.1764	− 0.6064	− 0.4659	185
2.0416	− 0.4516	+ 0.3373	197
2.0571	− 0.7121	− 0.0588	197
3.2971	− 0.7271	+ 0.2799	197
4.2481	− 0.7481	− 0.1652	197
2.353	+ 0.077	+ 0.3845	210

^1^“ + ” indicates that the measured value is overestimated from the true value.

^2^“−” indicates that the measured value is underestimated from the true value.

### Validation of remote sensing product with LAIS data

**Figure 9:**
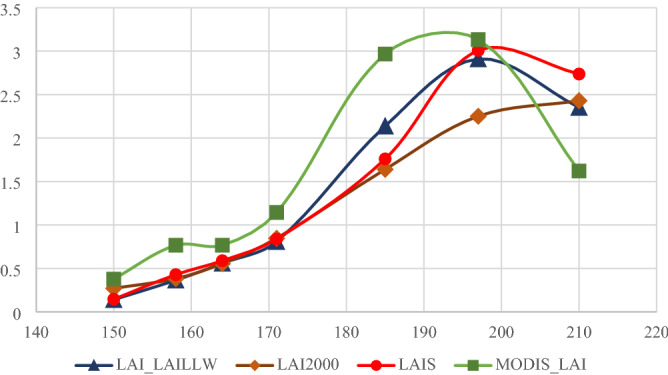
Variation curves of different LAI products in different observation periods.

We plotted the ground measurement data together with the large-scale MODIS LAI data in Fig. 9. We can see from the figure that the change curves of LAIS and LAI2000 almost coincide with the real leaf area index (LAI_LAILLW), while the MODIS data is higher than our real value. It is because the remote sensing pixel value is the average of the region, and the growth trend of corn is not the same in different plots of the region, and even there are other crops. So, the verification of remote sensing products is not easy to do. The more observation points on the ground, the better. WSN just provides this possibility. So, our work in the future is to make the LAI observation of WSN more convenient.

## Discussion

In this paper, based on the conventional LAI measurement method, a leaf area index (LAI) sensor which can automatically and continuously be monitored in real-time is developed, and two threshold algorithms are designed according to the growth stage of corn in different periods. Qu,y et al. also realized the automatic acquisition of leaf area index with the help of WSN system^17^, but they did not collect the image of the leaf, and the validation data is also the data of LAI2000 and did not use LAILLW (the true value of the LAI), so the results still need to be further verified. The measurement principle of our system is to obtain the image of the leaf itself, which is closer to the real value than the measurement principle of LAI2000, and the result also proves that this is the case. Similarly, Bauer, Jan et al. presented a modification of commercial PAR sensors^34^, which is also compared with the measurement data of LAI2200, but no image is used to obtain the leaf area. However, in our experiments, the leaf area index was calculated by image classification, which is more suitable for the small leaf of corn. The crop canopy images that were obtained when the leaves are small and the corn is sparse in the field were suitable for use with method 1 (“Image preprocessing and classification methods” Method1) .When the leaves were larger and they occluded each other, changes in the chlorophyll content and enhancement of the sun-light resulted in a low contrast between the leaf and the background in the true color images when the growth period reached two months into the panicle stage, it was no longer appropriate to use classification method 1, so we use method 2 (“Image preprocessing and classification methods” Method2).The improvement of image processing algorithm makes us get more accurate leaf area and character factor.

However, it is a disadvantage that human operation is needed to select the best quality image for classification. In the future, it is expected that this visual inspection process can be modeled to achieve full automation for data processing. Compared with the traditional field measurement methods, it is fast and accurate. The system based on a wireless sensor network not only saves time and labor but also increases the time and frequency of data acquisition, which can realize the detailed analysis of crop growth. At the same time, our verification results show that the measurement error of LAIS is less than LAI2000 in the early growth stage of corn. Although the result of our measurement method will be higher than the real value, it is due to the influence of weeds on the ground. When the LAI value is greater than 1, the measured value of LAI2000 is seriously underestimated, while the estimated value of LAIS is relatively stable. We also used LAI2200 in the experiment, but because LAI2000 was used twice, to keep the data consistent, we use LAI2000 to name them. Their measurement principle and accuracy are the same. Perhaps the new LAI instrument has been improved, but as a system that can automatically obtain crop images and monitor crop growth status in real-time, our measurement system and method have great advantages, because it not only obtains more detailed growth information but also saves manpower and material resources. What's more, our results are reliable, especially at the early stage of corn growth.

Another important issue is the bidirectional reflectance effect of vegetation canopy, which has a subtle impact on LAI estimation from satellite image^35,36^ and ground-based measurement as well. Part of the bidirectional reflectance effect comes from the solar illumination angle. The LAIS image taken at 1:30 p.m. is of a small solar zenith angle, the direct sunlight can better penetrate dense vegetation canopy to illuminate soil background; on the other hand, strong direct light may cause glare in leaf and become a disturbance in the classification process. So, when the vegetation is sparse, we prefer to use the LAIS image taken at 6:30 p.m. when the sky diffuse light dominates. Anyway, as the LAI estimation is based on the binary images as in Fig. 6, the effect of illumination angle will not affect the final result so long as the classification is successful. Another part of the bidirectional reflectance effect comes views angle. If the camera takes image off-nadir-view, then a view angle correction must be applied to the gap probability ${P}_{i}(\theta )$, and leaf angle distribution, which is represented by $G(\theta ),$ should be considered. Our strategy in this study is to fix the camera in nadir view, and use only the part of the image with a view angle less than 30°. However, the off-nadir-view image can also be used in LAI estimation. The optimal view angle and its directionality in LAI estimation should be discussed in our future research.

## Conclusions

The work in this paper improved the measurement of LAI to an extent and the data of the previously developed system is compared and verified. The system verification was performed by comparing the results with those from LAILLW measurements. A comparative analysis of measurement results from the LAIS and commonly used instrument LAI2000 was also performed. At the same time, using the data obtained by the LAIS to validate the remote sensing products of the leaf area index, it is concluded that the wireless sensor network monitoring system can provide test values for large-scale spatial data. Our data results are also true and reliable and can be applied to LAI inversion in the future.

This experiment improves the finite length average method, which improves the accuracy of the calculated LAI. The slope of the fitting line of the LAIS is 0.944, which displays a highly consistent with the true LAI that was acquired using the LAILLW method. The RMSE is 0.264 of the LAIS, smaller than that of LAI2000 (RMSE = 0.373). The absolute error is 0–0.6 and the relative error of most LAIS data is less than 30%. The classification results for the false-color image containing near-infrared information are better than those that are obtained from the true color image when the blades of corn are large and form the occlusion. The overestimation of LAI in the system is largely caused by the existence of grass. The strong sunlight is the main factor, reducing the discrimination between the soil and vegetation, and thus producing an underestimation when the leaves are in small size.

The outcome that the LAIS showed a better performance than LAI2000 supports its use to the application and popularization on farmland. With the verification of MODIS LAI, we conclude that the leaf area index measurement system based on WSN can provide test values for large-scale spatial remote sensing data. Of course, our system also needs to be improved. The further accuracy of the image recognition algorithm will have an important impact on the accuracy of the leaf area index.

## Supplementary Information

Supplementary Information 1.

Supplementary Information 2.

Supplementary Information 3.

Supplementary Information 4.
