# Supplementary material for: Validation of leaf area index measurement system based on wireless sensor network
Source: Sci Rep. 2022 Mar 18;12:4668. doi: 10.1038/s41598-022-08373-z (PMC8933413; doi:10.1038/s41598-022-08373-z)
Supplement: Supplementary file 1 — Supplementary Information 1. [file 41598_2022_8373_MOESM1_ESM.pdf]

GB2019 Leaf Area Index Remote Sensing Product Authenticity Test

1. Work profile

(1) Task source

In 2016, the National Standardization Management Committee approved the national standard setting project of "Leaf Area Index Remote Sensing Product Authenticity Inspection" (National Standards Committee Comprehensive [2016] No. 39), the standard plan project number is 20160472-T-491, and the Chinese Academy of Sciences is officially entrusted with remote sensing and The Digital Earth Institute takes the lead in completing the task of formulating this standard.

(2) Drafting unit and drafter

This standard was drafted by the Institute of Remote Sensing and Digital Earth, Chinese Academy of Sciences, Fuzhou University, Institute of Geographic Sciences and Natural Resources Research, Chinese Academy of Sciences, Peking University, Beijing Normal University, University of Chinese Academy of Sciences, Institute of Resource Information, Chinese Academy of Forestry, and China Agriculture Institute of Agricultural Resources and Agricultural Regional Planning, Academy of Sciences, Institute of Optoelectronics, Chinese Academy of Sciences.

The main drafters of this standard are: Li Jing, Zhao Jing, Zou Jie, Zeng Yelu, Liu Qinhuo, Fang Hongliang, Tang Bohui, Fan Wenjie, Qu Yonghua, Mu Xihan, Jiang Xiaoguang, Chen Erxue, Wu Wenbin, Jia Yuanyuan, Dong Yadong, Wang Xinhong, Liu Zhaoyan.

Drafters are responsible for the organization and coordination of standard setting work, the review and collection of related materials, the drafting and writing of standard texts and preparation instructions, organizing seminars, and collecting, sorting and summarizing relevant information through e-mail, fax, telephone, etc. Opinions and suggestions, as well as soliciting opinions within the industry and submitting standards for review, etc.

(3) Main work process

In January 2012, with the support of the National High-Tech Research and Development Program (863 Program), the standard specification research work of "Leaf Area Index Remote Sensing Product Authenticity Inspection" was launched, and a compilation team was established to clarify the scope of relevant research content and key nodes , Time planning and task division.

From February to August 2012, the collection of data and literature mainly included: collection of domestic and foreign standards and norms related to the authenticity of leaf area index remote sensing products, related books and literature, and field experiment reports.

From September 2012 to December 2012, the first draft was prepared. It mainly includes collating and summarizing relevant information; soliciting relevant expert opinions and suggestions, researching and determining the scope of the standard, and clarifying the standard name as "Leaf Area Index Remote Sensing Product Authenticity Inspection", determining the authenticity inspection standard catalog, and compiling the authenticity inspection related content.

From January 2013 to March 2014, exchanges within the group and unified opinions; draft preparation and research, listening to expert suggestions; soliciting expert opinions in related fields. The revision opinions not only come from the compiling unit (Institute of Remote Sensing and Digital Earth, Chinese Academy of Sciences, Fuzhou University, Institute of Geographic Sciences and Natural Resources Research, Chinese Academy of Sciences, Peking University, Beijing Normal University, University of Chinese Academy of Sciences, Institute of Resource Information, Chinese Academy of Forestry, The Institute of Agricultural Resources and Agricultural Regional Planning of the Chinese Academy of Agricultural Sciences also refers to the relevant observational norms of NASA, the School of Earth and Environment of Boston University, and the French Academy of Agricultural Sciences, as far as possible, to make up for the international deficiencies in this regard. The draft standard and proposal of "Leaf Area Index Remote Sensing Product Authenticity Inspection" were finally formed, and an application was submitted to the National Remote Sensing Technology Standardization Technical Committee (hereinafter referred to as the Remote Sensing Standardization Committee).

From April to October 2014, experts in related fields were widely solicited opinions on the revision of the first draft of the standard. The revision opinions mainly come from external scientific research institutes (Nanjing University, Cold and Arid Regions Environmental and Engineering Research Institute, Chinese Academy of Sciences, Qinghai-Tibet Plateau Research Institute, Chinese Academy of Sciences, etc.) that have not participated in the drafting of the standards. The revised standard draft was reviewed and approved by the members of the Remote Sensing Standardization Committee to recommend the project, and again based on the comments of the members, submitted to the Secretariat of the Remote Sensing Standardization Committee, and reported to the National Standards Committee to declare the project.

From September to October 2015, on the basis of the draft standard, we continued to solicit revision opinions from experts in related fields through a combination of standard draft seminars and expert communication consultations to form a revised draft standard and submit a remote sensing standard Secretariat of the Committee.

In November 2015, the National Standardization Management Committee reviewed the "Leaf Area Index Remote Sensing Product Authenticity Inspection" at the recommended national standard project evaluation meeting.

In June 2016, the National Standardization Management Committee approved the project.

From July 2016 to March 2018, further data research and internal exchanges were carried out, expert opinions were repeatedly sought, and the standard working group discussion draft and preparation instructions were written.

In April 2018, the remote sensing product authenticity inspection national standard specification seminar, after thorough discussion, clarified the remote sensing product authenticity inspection guidelines and the correlation between the general method of remote sensing product authenticity inspection and the standards, and standardizes each specific parameter standard Matters needing attention in the process of specification writing.

In May 2018, at the expert opinion meeting on national standards for authenticity inspection of remote sensing products, six experts participated in the meeting and proposed amendments to the rationality, standardization, and operability of the leaf area index product authenticity inspection plan. After the meeting, the drafting group further revised and improved the standard, formed the first draft of the standard for soliciting comments, and submitted it to the secretariat of the Remote Sensing Standards Committee.

From July to August 2018, in accordance with the revised opinions of the experts of the Standards Committee, the authenticity inspection plan of the leaf area index remote sensing products was further revised.

From October to December 2019, in accordance with the revised opinions of the experts of the Standards Committee, the authenticity inspection plan of the leaf area index remote sensing products was further improved, and the revised standard draft was formed.

2. Principles for the compilation of national standards and arguments for determining the main content of national standards

(1) General principles

The leaf area index remote sensing product is based on the spectral characteristics of vegetation and extracts leaf area index parameters from the reflectance of visible light and near-infrared bands observed by remote sensing. It is one of the basic parameters that characterize vegetation change information. Satellite remote sensing data are used at home and abroad to produce a number of leaf area index remote sensing products with different resolutions at global or regional scales. The differences in algorithms and temporal and spatial resolutions of each product make it difficult to obtain ground reference values in authenticity verification. In the acquisition of the ground truth value of the leaf area index, due to the different observation methods, the data is not comparable and difficult to be widely used; in the test method, different test methods such as direct comparison, averaging or scaling up are used to test. The results are also significantly different. In order to reduce uncertainty, it is urgent to formulate scientific and standardized inspection standards.

The general principle of the preparation of this standard is to standardize the inspection process and evaluation indicators of the authenticity inspection of the leaf area index remote sensing products. In-depth investigation and research and repeated review and revision are carried out to establish a scientific, standardized and operable leaf area index. Remote sensing product verification standards and specifications,

through a scientific and rigorous process to evaluate the uncertainty of leaf area index remote sensing products, to meet the development needs of leaf area index remote sensing product applications.

(2) Compilation principle

The preparation principles of this standard follow the principle of "clear definition, process standard, clear requirements, and feasible operation". The standard defines the concepts and terms in the authenticity inspection of leaf area index remote sensing products, standardizes the inspection process and evaluation system, and ensures the consistency of the authenticity inspection of the leaf area index remote sensing products, so that users can apply When the leaf area index remote sensing product, the uncertainty information of the leaf area index product can be accurately understood.

(3) Arguments for the main content of the standard

This standard specifies the ground sampling design, authenticity inspection method, evaluation index, etc. for authenticity inspection of leaf area index remote sensing products, and is used to guide the authenticity inspection of leaf area index remote sensing products. Regulatory documents include: "GB/T 3358.2-2009 Statistics Vocabulary and Symbols Part 2: Applied Statistics", "GB/T 14950-2009 Photogrammetry and Remote Sensing Terminology", "Guidelines for Authenticity Inspection of Remote Sensing Products", " General Methods for Authenticity Inspection of Land Quantitative Remote Sensing Products", and also refer to some of the latest research results in remote sensing authenticity inspection.

1). Terms and definitions

In the process of verifying the authenticity of the leaf area index remote sensing product, it involves important parameters such as the leaf area index remote sensing product to be tested, the true leaf area index, the effective leaf area index, and the aggregation index. The inspection process involves ground sampling, scale conversion, and The main process of authenticity inspection method, accuracy evaluation and so on. Therefore, the terms involved with strong professionalism need to be clear.

The terms and definitions used in the formulation of this standard mainly refer to the "Guidelines for the Authenticity Inspection of Remote Sensing Products" and the "General Methods for the Authenticity Inspection of Land Quantitative Remote Sensing Products" and the materials in frontier papers in related fields at home and abroad.

2). Inspection method

The authenticity inspection process of leaf area index remote sensing products is affected by many factors, such as ground observation data errors, inconsistent spatial scales of different products, and inconsistent synthesis cycles of different products. These factors have led to the need to clarify the sampling strategy and scale conversion of the ground observation data in the authenticity test of the leaf area index remote sensing product.

The main basis for the compilation of the remote sensing authenticity inspection method of the leaf area index is the "Guidelines for the Authenticity Inspection of Remote Sensing Products" and the "General Methods for the Authenticity Inspection

of Land Quantitative Remote Sensing Products", as well as the latest research results of the authenticity inspection of remote sensing products, such as surface reflections. Rate observation related specifications, research progress of remote sensing product authenticity verification methods for key land surface parameters, scale conversion methods, multi-scale verification strategies and uncertainty analysis, etc.

a. According to the "Guidelines for the Authenticity Inspection of Remote Sensing Products", combined with the main leaf area index product inspection methods at home and abroad, the leaf area index remote sensing product authenticity inspection methods are divided into two categories, direct inspection and indirect inspection, and a total of 3 recommended Testing method. Among them, direct inspection is an inspection method based on ground observation data; indirect inspection is divided into multi-scale step-by-level verification based on ground observation and high-resolution data, and cross-checking based on remote sensing products with known precision reference leaf area index.

b. Basic requirements, specifying the time and space distribution requirements and quality assessment of the verification data, as well as the description in the authenticity inspection report.

c. Sampling strategy for ground observation data, through the spatial representative evaluation of plots and the design of ground sampling methods, to obtain the relatively true value of the ground leaf area index that is uniform or that can represent the uneven surface area.

d. Scale conversion to solve the problem of representative differences between ground observation scale and satellite observation scale, high-resolution satellite data scale and low-resolution satellite data scale.

3). Precision index

According to the "Guidelines for the Authenticity Inspection of Remote Sensing Products", the important technical indicators in the standard reflect the temporal and spatial statistical characteristics of the leaf area index product and the verified relative true value to evaluate the accuracy of the leaf area index remote sensing product. The main evaluation indicators include coefficient of determination (R'), root mean square error (RMSE), average absolute error (AE), deviation (B) and product accuracy (PA).

4). The content of the inspection report

According to the "Guidelines for the Authenticity Inspection of Remote Sensing Products", the content of the inspection report specifies the information of the leaf area index product to be verified, the inspection method, the uncertainty analysis of the verification value, the evaluation conclusion and the production recommendation for the leaf area index product.

3. Technical demonstration of the main test (or verification) and expected economic effect

(1) Technical demonstration

During the preparation of this standard, full consideration was given to the

technical feasibility in scientific practice. According to the credibility of the reference relative truth value and the difficulty of obtaining it, a variety of inspection methods are given. Different inspection methods are suitable for different actual situations. The integration of multiple methods is not only helpful to ensure the reference to the data source of the relative truth value, but also helps to objectively understand the accuracy of the leaf area index product as a whole. The steps in the standard are organically connected around the core problems in the inspection, the solutions are comprehensive, and there are corresponding countermeasures for different problems, and the operability is strong. The inspection process is clear and can be continuously improved and supplemented on the existing basis, and it is extensible.

Various key technologies are considered in the preparation of the standard to improve the applicability of the standard. (1) Considering different surface types on a global scale, combining multiple methods, and comprehensively using multiple data sources to realize the accuracy evaluation of remote sensing products; (2) Considering the characteristics of vegetation time change, verify that the data contains at least one vegetation area on the time scale The growth cycle reflects the dynamic changes of vegetation growth.

The standard content organization follows a structure of consistent main lines, prioritized priorities, and complementary. The credibility of a variety of test methods ranges from high to low, the reference relative truth value is obtained from difficult to easy, and the scale of application is from small to large, which is suitable for different actual situations.

(2) Expected effect

This standard proposes the basic principles for the pixel-scale authenticity inspection of remote sensing leaf area index remote sensing products; it has formed an authenticity inspection method and system suitable for different scales of leaf area index remote sensing products; it can be used to guide different scales in global or local areas. Range of leaf area index remote sensing product inspection.

The implementation of this standard requires the standardization and standardization of global satellite leaf area index product verification, which facilitates the mutual comparison and conversion of different leaf area index products, and establishes a global leaf area index product with relatively consistent long-term series.

The remote sensing leaf area index product that has been verified for authenticity can reduce the uncertainty of the product itself. Studies have shown that after ground verification and calibration, the error of remote sensing leaf area index products can be significantly reduced.

Accurate uncertainty assessment will help promote the development of leaf area index remote sensing products, improve the ability of vegetation dynamic monitoring, mapping and resource management, thereby improving crop growth, pest monitoring and crop yield estimation efficiency.

4. The degree of adoption of international standards and foreign advanced standards, and comparison with similar international

and foreign standards

Regarding the authenticity test of satellite leaf area index products at home and abroad, at present, MODIS leaf area index products, BigFoot and VALERI projects have formed their own evaluation methods and indicators, but the standards and specifications are not mature. In recent years, a series of remote sensing product authenticity verification studies have been gradually carried out in China, and hydrological observation systems such as WATER and HIWATER in the Heihe River Basin of Gansu have been established. There are related documents that have simulated the scale effect of the leaf area index, but have not yet carried out specific research. The authenticity inspection work.

A comprehensive and reliable accuracy evaluation of leaf area index products requires standardized, standardized, and feasible technical processes to conduct joint and continuous authenticity checks in multiple regions and long-term series. Therefore, it is necessary to form relevant norms to facilitate the comprehensive utilization of the results. This standard refers to the successful experience of relevant research work at home and abroad, supplements feasible solutions for existing problems, and compiles it in accordance with different needs in practical applications.

5. Relationship with relevant current laws, regulations and mandatory national standards

The preparation of this standard refers to the content of some existing standards and is consistent with relevant current laws, regulations and mandatory national standards. The main reference standards are as follows:

1). National standard "Statistics Vocabulary and Symbols Part 2.: Applied Statistics" (GB/T3358.2-2009) and "Photogrammetry and Remote Sensing Terminology" (GB/T 14950-2009, which restricts the remote sensing terminology involved in this standard) .

2). Inherit the relevant provisions of the standard "Guidelines for the Authenticity Inspection of Remote Sensing Products" and "General Methods for the Authenticity Inspection of Land Quantitative Remote Sensing Products".

6. Process and basis for handling major divergent opinions

During the entire application and preparation period from January 2012 to December 2019, the drafting team solicited opinions from relevant research institutes, industry departments, and overseas experts in stages, and there was no major disagreement.

7. National standards as mandatory national standards or recommended national standards

It is recommended to be published as a recommended national standard.

8. Implementation of the requirements of national standards and

suggestions for measures

At present, China does not have a national standard for authenticity inspection of leaf area index remote sensing products. The establishment of this standard will directly guide the specific authenticity inspection process of leaf area index remote sensing products, and it is consistent with the authenticity inspection of leaf area index remote sensing products. Sexual expression has played a normative role, and it is recommended that this standard be released as soon as possible after approval.

9. Suggestions on abolishing existing relevant standards

without

10. Other matters that should be explained

For terms that are not specified in this standard, refer to the provisions of other industry regulations and standards.

"Leaf Area Index Remote Sensing Product Authenticity Inspection" Compiling Team

December 2019
