# Supplementary material for: Validation of leaf area index measurement system based on wireless sensor network
Source: Sci Rep. 2022 Mar 18;12:4668. doi: 10.1038/s41598-022-08373-z (PMC8933413; doi:10.1038/s41598-022-08373-z)
Supplement: Supplementary file 2 — Supplementary Information 2. [file 41598_2022_8373_MOESM2_ESM.pdf]

LAI-2000 PLANT CANOPY ANALYZER INSTRUTION MANUAL

Brochure Introduction

Chapters 1 and 2 learn the basics of instrumentation.
Chapter 3 guides the steps for simple measurements.
Chapters 4 and 5 detail the measurement of leaf area index and leaf density.
Chapter 6 Data transfer a data is transferred from the instrument to a printer or computer.
Chapter 7 describes the operating principles in detail.
Chapter 8 Instrument Maintenance.
Chapter 9 Introduction to console software features.
Chapter 10 Troubleshooting.

The appendices in this manual are important as a summary of the keys to the correct use and operation of the instrument. Specific operations are described in detail in each chapter and a brief summary is provided at the end of the manual.

1. Brief Introduction

The **LAI-2070** console includes the necessary electronic components to measure, record and calculate the final results. Powered by batteries, 64K storage space. There are two BNC connectors for connecting additional LI-COR light sensors (accessories) and two 15pinD connectors for connecting one or two LAI-2050 optical probes. RS-232 connector for transferring the recorded and calculated data to a computer or printer. The console is powered by a 6D battery and can be used continuously for 270 hours (approximately one month of continuous use, 8 hours per day). When the battery power drops to 15%, the screen will indicate that the battery is low (see chapter 8 for details).

Carrying case to carry the instrument on the shoulder or tied around the waist.

The **LAI-2050** light probe can be regarded as an expensive camera lens. The probe consists of a filter and a number of lenses which should be protected from sudden shocks or severe vibrations. Keep the exterior of the

lens clean and free from scratches. Damage to the lens is not covered by the warranty. See the warranty clause in chapter 8.

The lens cover is used to limit the azimuth of the probe in certain environments (see Chapter 4, summarized in Appendix G). The solid lens cover is used to protect the lens when the instrument is not in use. The lens is kept clean with a lens brush, cleaning solution and lens cleaning paper.
and drying.

Each light probe is equipped with two bubble levels, one of which is a bottom level to allow levelling when the probe is placed above the operator’s head.

Two **RS-232** cables are provided. The cable with the different concave and convex ends is used to connect the console to a Data Terminal Equipment (DTE) (e.g. a computer) and the cable with both convex ends is used to connect the console to a Data Communication Equipment (DCE) (e.g. another console).

The calibration sheets indicate the response of the optical probe at different angles in an isometric environment. Enter these five values into the console, see chapter 3.

The **1000-90 application** software is a program that downloads data from the LAI-2000 to the computer - **2000 - 90 The LAI-2000 DOS** software is another program (C2000) used to further analyses the data files. The C2000 program is used throughout this manual.

2. Basics

This chapter is a brief introduction to the principles and operation of the LAI-2000. 2.1 Introduction to the Principle

The amount of vegetation canopy foliage can be deduced by measuring how much light is weakened as it passes through the canopy. The LAI-2000 simultaneously measures the attenuation of diffuse sky rays at five angles. Thus, if the probes are oriented horizontally towards the sky, detector 1 will measure the brightness directly above and detector 5 will measure the brightness centered on the zenith at an angle of 68° radian at 13° (22° from the horizon).

The LAI-2000 measurements yielded at least 10 values: 5 values when the probe was above the canopy and 5 values when the probe was below the canopy. In both measurements, the probe was always pointing towards the sky. The 5 values of canopy transmittance are obtained by dividing these corresponding readings by two, e.g. if the reading of detector 1 above the canopy is 50 (in insignificant units) and the reading below the canopy is 5, then the

transmittance at this angle (central angle of 7.) is 5/50 = 0.10. From these 5 zenith angle transmittances, the LAI-2000 can calculate the number of leaves (leaf area index, LAI) and the leaf tilt (LAI). LAI) and leaf tilt (mean leaf tilt angle, MTA). o In practice, values under several canopies are often taken for spatial averaging.

2.2 Assuming ploughing

In order to correctly calculate the number of blades and the tilt, a number of assumptions must be made, the extent to which they are involved will affect the confidence of the results. There are four main assumptions, listed in order of importance, as follows.

The leaves are opaque and non-reflective. The LAI-2050 probe has a light filter which does not accept light greater than 490nm. Also, the leaves, it also rarely reflects or transmits light in the blue region of the spectrum.

The arrangement of the leaves is random. Different canopies have different shapes, which may be parallel tubes (strip crops), ellipses (solitary trees), very large boxes (grasslands) or large boxes with holes (deciduous forests with gaps in the forest). The distribution of leaves in these 'vessels' is random.

The vane area is small in relation to the observation range per ring. This relationship is quantified in Appendix F and can also be roughly estimated: i.e. the distance between the probe and the nearest blade should be at least 4 times the blade width.

The blade azimuth is random. This means that all blades do not face the same angle, regardless of how they are tilted. This assumption does not matter when measuring with a wide angle of view.

None of the canopies fit these assumptions perfectly. The leaves are never randomly arranged, but are clustered on stems or branches. The fact that many plants exhibit unwariness also reduces the assumption of random orientation. However, this necessary assumption is not really very important. Many canopies can be considered to be randomly distributed, and leaves do have relatively low reflectance and transmittance for light below 490 nm. Errors often offset each other, e.g. when leaves are clustered on branches (increasing transmittance), but arranged in such a way that they do not block each other as much as possible (decreasing transmittance).

There are also cases where there are large deviations from the ideal, such as conifers (discussed in Chapter 4) with highly organized leaves, or senescent crops with fine reflective leaves. In these cases, the absolute

errors in LAI can be large and the only solution is to look at their relative differences or to calibrate the instrument with direct measurements.

2.3 Operation Forest

The LAI-2000 requires measurements above and below the canopy for its calculations, which can be done in three ways.

One probe approach: both the upper canopy reading (A) and the lower canopy reading (B) are taken with a single probe, this approach is suitable for short canopies where the probe can easily be placed on the canopy. This measurement starts with the A reading and when the B reading is recorded, the instrument uses the nearest A value to calculate the transmittance. If the sky conditions are stable, one set of A values can be applied to several sets of B values. If sky conditions are changing rapidly, a new set of A values should be taken quickly before each set of B values.

Two probe approach: both probes are connected to the mainframe, one above the canopy and one below the canopy, and both sets of values are recorded and the transmittance is calculated. It should be noted that if both probes are placed under the same conditions they should give the same reading and the mainframe has a calibration procedure which should be self-calibrated before measurement. This approach is suitable for tall canopies and also addresses situations where sky conditions can change rapidly.

The remote sensing method: one probe and the mainframe automatically measure the A-value (e.g. every 15 seconds) and the other probe and mainframe manually measure the B-value, with the same calibration as the two-probe method. The two hosts are then connected and the B-value host automatically finds the required A-value and calculates the transmittance, or the data can be transferred to DOS and combined using the C2000 program.

2.4 A simple Hirsch file

After the measurement, the data is stored in a 16K memory in the mainframe. When the last value has been recorded, the LAI and other parameters are calculated and all observations and calculated values are stored in a 64K main memory in the form of a file, which can be Display of files on the host computer or output of files in standard or user-defined formats (RS-232).

FILE	DATE	TIME	CROP	PLOT	LAI	SEL	DIFN	MTA	SEM	SMP
19	14 APR	19:47:26	WHEAT	5	1.00	0.07	0.472	64	1	15

ANGLES	7.000	23.00	38.00	53.00	68.00
CNTCT#	0.322	0.367	0.491	0.543	0.510
STDDEV	0.204	0.214	0.208	0.155	0.092
DISTS	1.008	1.087	1.270	1.662	2.670
GAPS	0.723	0.671	0.536	0.406	0.256

A	1	19:47:36	4.101	4.507	5.420	6.842	8.058
B	2	19:47:46	3.750	3.881	3.867	3.404	2.352

:

The document has three parts: the header, the statistics and the observations.

The first two lines of the file are the header, which includes the file number (set by the system), the time the file was generated, a note from the user, and the results of the calculations: Leaf Area Index (LAI), Standard Error of Leaf Area Index (SEL), Scattering Without Interception (DIFN, the portion of the sky visible to the probe), Mean Inclination Angle (MTA), Standard Error of Mean Inclination Angle (SEM), and the number of sets of A and B observations used in the calculations.

The set of statistical values consists of five sets of data containing five numbers: ANGLES is the center angle value of each of the five rings, CNTCT# is the mean contact frequency [Iog(gap/path length)], STDDEV is the standard error of contact frequency, DISTS is the path length, and GAPS is the gap fraction. These statistics and how the values in the header were calculated are discussed in Chapter 7. For a fully covered canopy, DISTS is 1/cosΘ. When measuring a single plant, these values should reflect the actual path length observed in the canopy for each ring of the probe. This method of measurement is discussed in detail in Chapter 5.

The observations section includes the "A" and "B" marks, the sequence number, the recording time and the value of the probe's 5 rings.

The numerical meaning: LAI answers the question "how many leaves are there" and although LAI literally means "leaf area index", the LAI-2000 measures all objects that block light. However, if DISTS is not 1/cos angle, then LAI is the leaf density (mf, see Chapter 5).

The **MTA** answers the question "How tilted are the blades?" If all blades are horizontal, then the MTA is 0° : if all are vertical, then 90° ° - generally the MTA is between 30° (horizontal blades dominate) and 60°

(vertical blades dominate). If an unintended MTA value occurs, this indicates a measurement error or an incorrect DISTS value, see chapter 10. For further information on the distribution of tilt, the C2000 program can be used for analysis.

DIFN is calculated by combining the gap fraction (**GAPS)** to represent the part of the sky that is not shaded by foliage. This value ranges from 0 (full foliage) to 1 (no foliage) and DIFNS can be broadly considered as a proxy for canopy structure, combining LAI and MTA into one value. Of all the values calculated by the LAI-2000, DIFN is the most indicative of 'canopy light absorption'. However, canopy absorption is not only related to the structure of the canopy (DIFN), but also to the light characteristics of the leaf in the available light range (e.g. photosynthesis is only effective for 400-700 nm light), the ground surface under the canopy and the position of the sun. The DIFN is therefore only an indicator of the absorption of short-wave scattered rays (less than 490 nm) by the canopy.

Explanation of this example: The LAI of winter wheat observed in the evening of 14 April was 1.00, with a mean tilt angle of 64° , and about 47% of the sky visible under the canopy, which was very heterogeneous, as the standard deviation was 0.27 (SELX JSMP). However, the large sample (15) reduced the standard error, so it can be said that the mean LAI was 1.00 ± 0.15 (SELXtog i.e. 0.07X2.145) at the 95% confidence limit.

Comparing the three sets of A values (1, 7 and 13) gives an idea of the sky conditions and their stability: we find that the part of the sky close to the horizontal angle is the brightest and gradually gets darker within 2 minutes.

Note: Checking the A-values can reveal problems. If the A-values are inconsistent, this indicates poor measurement technique or poor sky conditions. If there is a large difference between the values in a set of A values, the sun is not blocked or the battery is faulty or the calibration values are wrong. This is described in Chapter 10.

3. Getting to know the instruments

1. Connections
2. Software

An LAI-2000 mainframe and an LAI-2050 probe are applied to perform the following operations.

- The calibration value of the input light probe.

- Set time

-Input measurement content

- Two prompt questions in the definition notes
- Take measurements

-Survey data

The following measurement steps can be simulated in the room.

(1) Connect the **LAI-2050** probe to the X-port, with the X-port on the left and the Y-port on the right when looking directly at the panel.

(2) Turn on the power switch. Press the ON button (top right corner) to switch on the power. 2 seconds later the screen displays the software version number and its date. Press FCT09 to switch off the power. If no key is pressed within 1 hour, the power will be automatically switched off.

(3) Perform the setup operation. Press SETUP (top left) and press the arrow keys to select the menu

00 **Set

01 XCal

02 Y Cal 03

Vectors

04 Resolution

05 Set Clock

06 Set Dists

07 Set Angles

08 1,2Channels

09 Off

Simply place the desired entry on the first line of the display bar and press ENTER to run it. You can also run it by pressing FCT+code. We will only set 01, 04 and 05, the rest will still use the default values.

Select this item and enter the 5 values listed on the light probe calibration sheet. If the probe is connected to In the Y position, then item FCT 02 should be selected to enter the correction value.

Each key has a blue letter in the top left corner, press the up arrow key and then press the key where the letter is to enter it, if you do not press the up arrow key you will enter the number shown in the key. To modify, use the left arrow key. If you do not enter any value and press the Enter key, the setting remains the same. Press the BREAK key to interrupt the

programmer at any time.

4 **Resolution.** To enter this item, press the up and down arrow keys to select the different settings, there are two options, high precision and low precision.

5 **Set Clock** Enter this item, enter the corresponding value and press the right arrow to move it, the month is the first three letters, if you enter it wrong, the month column will be displayed as XXX.

(4) Set the mode of operation. Press OPEN to enter this menu. The list is as follows.

10**Oper
11 Set Op Mode
12 Set Prompts
13 Def Log Key
14 Log New
15 Log Append
16 Bad Reading
We perform FCT

After entering **Set OD Mode**, use the up and down arrow keys to select the measurement mode, select **1 sensor X and press** enter, two options will appear: one is the measurement order, the up arrow key represents the value above the canopy, the down arrow key represents the value below the canopy, if you want to set the measurement to **1 A** value and then **4 B** values, enter one up arrow key and four down arrow keys, press enter: the other is the number of repetitions. If you want to repeat the measurement 2 times, enter the value 2, then press the Enter key.

Set Prompts provides the user with a record of notes, or WHAT and WHERE if you wish to set two questions to sample name and sample location.

(5) Observation mode: simultaneous observation. Press BREAK for simultaneous observation, use the up and down arrow keys Make upward elections, left and right arrow keys make downward selections.

(6) Data logging and LAI calculation. Press LOG to start the data logging procedure. First enter the WHAT and WHERE values (up to 7 letters), then the status bar appears. The top row is the synchronous observation column and the bottom row is the summary column in the format Ordinal number of A values (B values are indicated when this column is in the lower row) * Ring number value Logarithm of accepted ABs (SMP) Average LAI ± SEL Hu. When recording press the Enter key, one sound is made and another sound is made after the data has been obtained, making sure that the probe is held in a horizontal position between the two sounds.

Press ENTER to record the first A value, then the synchronized

observation bar moves to the bottom row to indicate the start of recording the B value, and when the first B value is obtained, the calculated value is displayed at LAI. After four consecutive B values have been taken according to the set procedure, the synchronous observation bar returns to the upper row to start recording the next A value and then four more B values. At the end of the recording, the instrument calculates automatically.

(7) View the measurement results. The file is automatically saved after recording and can be viewed by pressing FCT 27. The file view format has 5 sections (view by numbers 1-5).

List 1: Title headers, remarks and results. The detailed list is as follows.

Document number
Date of document generation
Note 1
Note 2
LAI
SEL
DIFN
MTA SEN
Logarithm of AB values used for calculation
Another LAI value (calculated using the Lang method)

list 2: ANGLES and DISTS values, as follows. ANGLE and DIST values, document number

Value of ring 1
Value of ring 2
Values for ring 3
Value of ring 4
Value of ring 5

list 3: CNTCT and STDDEV values in the format of list2
list 4: WANGLES and GAPS values in the format of lisl2.
list 5: Observations.

4. Measurement

1. Probe correction

With the probe inserted at X, press FCT01 and enter 5 correction values: 4014, 1251, 1000, 1003, 1294. These correction values are relative to the individual rings and cannot be considered to be the energy value received by the probe. When calculating the LAI using the same probe to measure the AB

value, this correction is not very meaningful as the correction value is removed from the calculation.

This correction is essential when measuring with two probes.

The factory calibration values are measured without the mirror cover and may not be appropriate when the cover is used. In this case, an isometric correction should be made.

2. Application of two sensors (omitted)

3. Wrong reading value

In theory, the B reading should always be lower than the A reading. Under very sparse canopies the two values are close and in large forest gaps without leaves the two values agree, but this is only theoretical. In practice, the B reading is often higher than the A reading for several reasons.

- Changing sky conditions
- Normal operating changes
- Operator error (reversal of A and B sequence)
- B-value measured when blade reflection is strong (also operator error)

If one of the five readings in B is higher than the previous corresponding A value, this is also wrong and the resulting extrapolated transmittance will be greater than 1-0. If this is due to sky conditions or human factors, it can be corrected and measured again, but if it is due to sparse foliage and normal fluctuations, then it is best to ignore this difference and treat the transmittance of the ring in question as 1-0.

FCT 16 (False Readings) allows the host computer to adjust for values of transmittance greater than 1.0 during data recording and data file recalculation with three options: ignore, set **A/B=l**, set **B/A=l"**

- Ignore: When the transmittance is greater than 1.0, the instrument will chirp for about half a second after completing the recording of the value. This set of B values is not used in the calculation but is still present in the file. This set of B values is not used in the calculation, but is still in the file. It chirps once for each incorrect value in the recalculation (FCT26). This method is used when the actual values of A and B are not very close to each other.
- Let A/B = l: if the transmittance is greater than 1.0, this is considered to be 1.0o This does not change the stored data and is suitable when reading B values under canopies with little or no foliage.
- Set B/A=l: As with A/B=l, the difference is that the A value is assumed to be the sub-crown value and the B value is the supra-crown value. This should only be used in the "two-sensor approach", where the X-sensor is located under the crown and the Y-sensor is located above the crown. The

data collected can be calculated in one of the ways set in the FCT16, or it can be recalculated in another way, either with A/B=l, or B/A=l, the LAI is 0.

4. Actual assault

When sky and/or canopy conditions are not ideal, in most cases a modification of the measurement programmer is required, which is the main focus of this section.

When measuring, it is first necessary to clarify 'what to measure', in particular whether to measure the average LA1 over a large area or the leaf density of a single plant. Chapter 5 describes the relationship between the two and how to measure the leaf density of a single plant. Many of the practical considerations in this chapter also apply to the measurement of leaf density.

How much B value to measure. Firstly, determine the valid range of LAI values derived from the measured lot: the whole lot, a part of the lot or a small point? Secondly, which part of the lot does a B-value represent? As a rule of thumb, each B value represents the extent of the sample as a cylinder with a radius of the measured canopy height (considered as part of the cylinder when covered by a mirror). This gives

$$A = f\pi H^2 \qquad (4\text{-}1)$$

Where A is the sample area, f is the window range (0.75, 0.5, 0.25 and 0.125 for 270°, 180°, 90° and 45° covers respectively) and H is the canopy height. For example, a B value is taken from a canopy with a side length of 5m and a height of I am without a mirror cover (f of 1), which represents a sampling area of 3n? If the canopy is only 0.2m high, then the B value represents only 0.5% of the full area, so the height of the canopy should be considered when measuring.

Another note is the variation in leaf density at the point of measurement. It is sufficient to measure a smaller number of B-values in a homogeneous section compared to an inhomogeneous section. Appendix E describes how to determine the appropriate number of B-values on the basis of predicted quantities. For example, if the LAI = 2.0 and the standard error SEL = 0.1 are calculated on the basis of 6 B-values, then the actual measurement should be increased to 8 B-values in order to achieve a 95% confidence limit.

Measuring Position. Similar to the previous 'how much to measure' question, one solution would be to divide the canopy by one or shallow. Another method

would be to set the points at random, which would probably have a large error.

The position of the B-value measurement is often rarely changed to avoid a drifting blade suddenly blocking the probe during the measurement, Appendix F provides some minimum distance routes and the theory for their determination.

Foreign bodies in the visual field

When the experimenter is within the field of view, use the 270° mirror cover to shield the experimenter. If the experimenter is careful to keep himself in the same part of the field of view when measuring the AB value, the cover can also be used without the cover.

Usually, the presence of objects in the measurement of AB does not affect the calculation of LAI. If the AB values are very close to each other, the forest canopy is in the same part of the probe's field of view for the AB measurement.

Declare sunlight

Although direct sunlight is to be avoided, measurements can be taken on cloudy days or at sunrise and sunset. If measurements must be taken in direct sunlight, the following steps should be observed.

Cover the sun with a 270° lens when the sky is clear, or a 180°

lens if the sky around the sun is also bright.

Measure the AB value with your back to the sun and cover the operator and the sun with a mirror cover.

When measuring AB, even though the mirror cover is blocking the sun, the probe should be blocked with your hand or something else to avoid reflection.

The rays affect the values.

The more reflective foliage there is within the probe area, the smaller the LAI value will be.

LAI values will vary over time if the canopy is not shaded.

-Dispersion Cloud

If possible, measurements are taken when the sun is obscured by clouds, which can shorten the measurement time between AB values if the clouds are moving quickly.

-Changing overcast sky

If the sky has distinct bright and dark areas, errors are likely to occur when measuring uneven canopies: the canopy under the brightest direction of the sky will add more to the measurement. The solution is to reduce the field of view of the probe so that bright and shaded areas do not appear in the same reading at the same time.

Choose a suitable mirror cover to reduce the range of view of the probe

and maximize the range while ensuring. The maximum zenith angle seen by the probe is 74°, tan (74) = 3.48. In practice this is often taken as 3, which reduces the influence of the edge blades on the probe.

In this way, when the canopy is 1 meter high, the probe should have a visual range of at least 3 meters in any direction. If the sample point is so small that the required area cannot be measured when the probe is positioned in the center, a 90° mirror cover should be used to measure at the corners of the sample point. Under dense canopies, the minimum visible area of the probe is reduced due to the shading effect of the foliage.

-Slope

When measuring on slopes, it is important to keep the AB values measured at the same angle and in the same direction.

-Gaps in the canopy

The LAI is proportional to the logarithm of the gap section, so the correct average of the gaps is the average of their logarithms. This is in fact what the LAI-2000 does if it averages the B-values. However, any B-value is a linear average of any azimuthal rays measured through the mirror cover within the field of view of the probe, and an erroneous value will occur when recording B-values where there is a very dense canopy in one direction and no or very few leaves in another direction within the field of view of the probe. This is because voids in the canopy will be over-measured and LAI values will be under-occupied. The solution is to take a closer look at the

The canopy void test (Appendix D) can be used to measure this particular canopy when, with the application of a large-view mirror cover, the void and the leaf appear in the same B value, creating an underestimation error.

-Aligned crops. This technique is a good way of measuring the canopy of row crops from sparse to dense. The first B value is taken 1/4 of the way up the monk, the third is taken in the middle of the monk and the fourth is taken 3/4 of the way up the monk. This oblique sampling method shows a more uniform spatial (Appendix D), if the distribution than the straight sampling method.

Pre-history

If the error is greater than 10% (uneven canopy), a 45° lens should be used and the number of samples should be increased by at least two times, and a paired sample line should be used, i.e. the first sample line should be measured with the angle of view parallel to the monopoly, and the next sample line should be measured with the angle of view perpendicular to the monopoly.

-Rain, fog and 18

Small droplets of water on the probe can affect the results and care should be taken not to allow water to fall on the probe when measuring under

wet canopies. Measurements can be made in the rain as long as the probe is kept dry, but there is a significant degree of unreliability. The LAI-2000 assumes that the position of the leaves is random, so that the LAI values measured in the conifer canopy are low. A correction factor, R, is needed to modify this, and the authors measured R values of 1.49, 1.50, 1.67 and 1.60 for European larch, American red pine (Pinus resinous), five- needle pine (Pinus strobes) and Norway spruce (Pica abides), respectively. The LAI values obtained with this correction factor correlated strongly with the directly measured values (oven=0.96, $P<0.005$, n=5).

R = projected area of needles; average projected area of branches.

The LAI value measured by the LAI-2000 can be considered as the average branch projection area, the surface area of the needles is about three times the projection area of the needles, as the needles are different in shape from the cross-sectional glance, if the cross-section is cylindrical, the surface area of the needles is 3.14 times the projection area: if the cross-section is semi-cylindrical, it is 2.57 times; if the cross-section is flat, it is 2 times

Summary: The LAI-2000 measures the projected area of the branches in the needle leaves. The Needle Projection Area Index can be obtained as the product of the R-value and the projected area of the branches. To calculate the total surface area of the needles, the projected area index is multiplied by the appropriate conversion factor for the different cross-sectional shapes of the needles.

- High canopy and forest

The biggest challenge when measuring this canopy is the measurement of A-values. The values above the canopy can be easily obtained using remote sensing: one instrument is positioned on or outside the canopy to automatically record the A-value and the other instrument collects the B-value. The two instruments are then connected and the instrument reading the B value automatically goes to the appropriate A value for calculation.

Old +| * Pine

The one probe approach can also be effective for such measurements, especially with the help of the C2000 analysis software for adding A-values. The steps are as follows.

Each measurement starts and ends with the A value top moan large. -fruit sound

Enter the data into the computer and apply the C2000 software to insert the A-value. A-value insertion is carried out in the Compute Options command

line. The two A-values measured for each ring have a linear regression and a suitable A-value can be selected for the B-value.

Where to measure A values? The maximum distance at which the A Cook measurement point should be acceptable should depends on the sky conditions. In cloudless, clear skies, even if the AB points are kilometers apart, they will be in the same bright sky. Uneven, low clouds and fast moving skies are the least effective, and a distance of 100 meters may be too great.

If measuring A values in a forest clearing, ensure that the clearing area is large enough. When using a cover with a 180° or larger window, the probe is placed in the center of the clearing (precisely the gap area of the forest canopy) at a distance of 7 times (2 firetan74°) the diameter of the probe to the canopy, while with a 90 or 4 5° cover only half the area is needed. This is why narrow window covers are used in forests. If the clearing area is not large enough, the value of the outer ring can be ignored and the measurement can be made with a 90° or 4 5° lens cover in an area with a radius of 1.5 times the height of the tree.

Note: When measuring the A and B values, it is important to use the same mirror cover, the same angle of inclination of the probe (if the ground is sloping) and the same direction of observation.

5. Measuring single pygmy plants

This chapter discusses how to measure a single plant, which is completely different from the measurement of a mature canopy. It starts with the leaf area index and leaf density.

1. Leaf area index or leaf density

LAI is the area of leaves per unit area of ground. In a homogeneous plant community, LAI is a good representation of vegetation density. This is not the case for individual plants, as the density of leaves on a given ground is determined by the position of the ground, i.e. the density of leaves at the centre of the tree is different from the density of leaves at the edge of the tree. Also, the area of the ground affects the LAI value, whether the ground is based on the same projected area as the canopy or on the average growth area occupied by the tree. This makes the LAI of a single plant ambiguous, unless both the location and the area of the ground are given.

The leaf area density (or simple leaf density) method is an effective way of measuring the number of leaves on a single plant or clump, by removing

the leaf area from the volume of the canopy. This gives a unit of leaf density thus in a 5n? canopy with 2n? leaves, the leaf density is 0.4mJ

The principle of measuring the different LAI and blade densities, mainly the distance vector, is explained in Chapter 7, Introduction to Principles.

2. Distance vector

The instrument has a set of vectors (with 5 values) for calculation. These values indicate the distance of light through the canopy to the 5 different detection rings on the probe. They are presented in the file as DISTS and the default value is l/cos (.), 0 is the angular value for each ring (7°, 23°, 3° 8, 53°, 68°). There is no need to adjust these values for LAI measurements, as 1/cos (0) is always valid, regardless of the height of the canopy.

The exact mean path value for each ring through the canopy must be entered in the leaf density measurement and the resulting LAI value is the leaf density value. The units of leaf density are related to the units of the DISTS value, if the distance is expressed in m, then the units of leaf density.

3. A simple example

A shrub canopy is assumed to be a standard semicircle with a radius of 0.7 m. A semicircle canopy is the simplest because the paths of each ring are equal. To measure the leaf density of this shrub, first set FCT06, select Edit) and set each ring to 0.7. It is not possible to average the values spatially across the canopy, but only to take a B value at the centre, otherwise the measurements are the same as for LAI.

4. Isolated Trees

When measuring the crown of a tree it is best to use a cover of 180° or smaller and to place the probe close to the trunk, avoiding large branches in the line of sight. If there are no leaves in the outer rings, the C2000 can be used to remove these rings.

The 90° and 45° covers reduce the measuring area even further. Care should be taken to avoid adjacent canopies entering the viewport when taking measurements. If the canopy is balanced, several B-values can be measured in different directions and the data stored in one file: if the canopy is not balanced, several files must be used for storage, as the distance vectors are different in each direction.

The C2000 program is used to obtain path lengths and canopy volumes. Using the center of the lower edge of the canopy as the coordinate system,

the C2000 measures a sufficient number of points in coordinates to describe the shape of the canopy, and from these coordinates the C2000 determines the path length and the ring values to be ignored, and calculates the canopy volume, leaf density and drip line LAE

5. C2000 Example

The following are the steps when applying the C2000 to a single tree crown.

(1) This data is taken with a 90. mirror cap in each of 4 different directions for a pair of AB values.

(2) Eight pairs of XY values were measured to describe the canopy profile.

(3) Enter the file into the computer.

(4) Start the C2000.Read the file.

(5) The calculation is carried out using the Edit Canopy-model Individual line.8 The coordinate values are entered into the corresponding question prompt and the height of the probe from the ground. The program then automatically calculates the canopy volume, drip line area and path length (D1STS).

(6) Execute the Compute command and perform the calculation.

(7) Execute the Print Standard command to output the file in a standard format.

Leaf density is still labelled with LAI, where

DLLAI = leaf density X canopy volume drip line area.

SNRHGT is the distance of the probe from the ground, VOLUME is the volume of the canopy calculated from the canopy profile coordinates, and AREA is the drip line area under the tree calculated from the maximum canopy radius in the canopy profile coordinate system.

6. Data Transfer

The data stored in the LAI-2000 can be transferred to a computer or printer via the RS-232 data transfer line. This is described in detail in chapter 9.

1. planing port

FCT31 sets baud rate, data bits, parity and handshakingof the RS-232 port

2. Applications

There are two prerequisites for transferring data to a computer: one is

that an RS-232 port must be available and the other is that an executable program is required. These programs are available on disk number 1000-90. The procedure is roughly as follows.

- Connect the computer to the LAI-2000.
- Start the computer program.
- Verify that the computer is configured identically to the RS-232 port.

-Select the data storage location for input to the computer.

- Define the output format (FCT33), then transfer the file (FCT32).

3. Output file format

The FCT33 can define two output file formats: standard and extended.

- Standard, this format can be applied to the C2000 analysis software for further analysis.
- Extended, each file is displayed on one line only, with output for the column selected by the user, each column having a fixed code, see Chapter 9 for a detailed description.

4. 1000-90 and **2000-90**

The C1000 is file transfer software and the C2000 is file analysis software, both operating under DOS.

COMM in the C1000 is the execution of commands for file transfer.

C2000 is software for analysing, editing and recalculating files entered and saved in a standard format. Versatile analysis can be performed on one or more files.

- Print documents in standard or user format.
- Edit the document table header.
- Edit record values. Combine AB values from within different files into one file.
- The single plant canopy pattern is applied to the data file and DISTS is automatically calculated from the canopy dimensions entered by the user as discussed in detail in Chapter 5.
- Select a ring value that can be ignored.
- It is possible to recalculate using the same scheme as in the console, or to add other options to the calculation, such as adding upper canopy values or making the transmittance not exceed 1 (e.g. FCT16).
- Values not available in the console can be calculated, e.g. LAI and LAD (leaf area distribution) values for leaf angle classes 5 and above by constrained least squares inversion (Norman & Campbell, 1989): LAD by two-factor Beta distribution (Goel & Strebel, 1984): LAD and LAI by single-factor Campbell] 1986): canopy volume and drop

line LAL by single-factor Campbell] 1986). amp;Strebel, 1984): calculation of LAD and LAI using the single-factor Campbell's LAD calculation method: calculation of canopy volume and drop line LAL for single canopies

7. Principles

The LAI-2000 measures the sky as seen through the vegetation canopy in all directions. These values include two main aspects of canopy structure: the number of leaves and the tilt of the leaves. This chapter describes in detail how the LAI-2000 represents this information in terms of quantitative values.

1. Blade

When a beam of light passes through a certain thickness of vegetation canopy, the light changes due to the blocking effect of the leaves. This
The variation is proportional to canopy thickness, leaf density (the number of leaves per unit of canopy volume) and leaf tilt. If one assumes that the leaves are small in relation to the large canopy and are randomly distributed in the area through which the light passes, then one can know the variation from the angle (0)(is the zenith angle, (P is the azimuth angle) and the remaining light after passing through the canopy is

$$T(\theta, \varphi) = \exp[-G(\theta, \varphi)\mu S(\theta, \varphi)] \qquad (7\text{-}1)$$

where G(6 , 0) is the projected fraction of the leaf at the angle of light (, 9), the projected portion of the leaf under the angle of light, the news is the leaf density (area of the leaf per m of [3] canopy) and S(G , cp) is the distance of the canopy penetrated. Since the LAI-2000 probe section is averaged for any orientation, the azimuth 9 can be rounded off, i.e. it is understood that the variation values are averaged over each orientation. In this way, Equation 7-1 can be transformed into

$$G(\theta)\mu = -[\ln(T(\theta))]/S(\theta) = K(\theta) \qquad (7\text{-}2)$$

K(0) is the contact frequency (Miller 1967). This is the average number of contacts per unit penetration distance as the probe passes through the canopy. From Eq. 7-2, the press value can be derived

$$\mu = 2\int_0^{\pi/2} \frac{-\ln(T(\theta))}{S(\theta)} \sin\theta \, d\theta \qquad (7\text{-}3)$$

In a homogeneous full-cover canopy, leaf density and leaf area index L

are related to canopy height Z, and path S is related to canopy height and zenith angle. Related to.

$$L = \mu Z \qquad (7\text{-}4)$$

$$S(\theta) = Z/\cos\theta \qquad (7\text{-}5)$$

$$L = 2\int_0^{\pi/2} -\ln(T(\theta))\cos\theta\,\sin\theta\,d\theta \qquad (7\text{-}6)$$

Taking these into equation 7-3 gives LAI.

Because canopy height is eliminated in Eq. = -l/cose, then it is the same as Eq. 7-3. Thus, when the distance is -l/cose, the measurement is LAI: otherwise it is leaf density.

The LAI-2000 has 5 zenith angle values. They appear after ANGLES in the standard format file. The canopy non-intercept number T (0) is calculated from each pair of AB values and the logarithm of these numbers divided by the distance Si (i.e. DISTS) is the contact frequency K (0 i), for each zenith angle. i has an average K (G i) value and these average K (6 i) values appear in the file as CNTCT#, the standard error of each average is STDDEV, GAPS is the average transmittance Ti° obtained by substituting Ki values into Eq. 7-2. GAPS is the average transmittance Ti° obtained by substituting Ki values into Eq. 7-2 LAI is either the leaf area index or the leaf density, depending on the path length.

We have found that in canopies where the leaves are vertical (e.g. dwarf grass), scattered light makes the outermost ring readings smaller, so that the LAI values also become smaller, usually less than 10%. LAI can be avoided by applying the Lang method to calculate.

LAI=2 (Slp+Int) (7-8)

Sip and Int are the zenith angles. The lower slope and average number of contacts are intercepted, the LAI-2000 also performs this calculation but does not display it in the standard output format, this value can be viewed in FCT27 as 2* (S+I). It can also be displayed in a user set format.

2. Blade orientation

Once the leaf density or LAI value has been determined, the relationship between G (0) and leaf tilt can be obtained from Equation 7-2. The relationship between

The LAI-2000 calculation is based on Lang's (1986) method of averaging the body tilt angle MTA, which gives the average slope of an ideal G curve and a polynomial equation, which gives an empirical equation for the G slope

with respect to the blade tilt angle Once the LAI values are known, remove the contact values to 5 values using LAI to Apply the slope of the G (0) line to equation 7-9 to obtain the MTAo Add the standard error of the slope to the slope value, apply it again to equation 7-9 and subtract the MTA from the second angle of inclination, thus obtaining the standard error of the MTA SEMo **3g** stay **M**

A quantity used to characterise the radiation environment at a point within the vegetation canopy is the non-intercepted diffuse emission (DIFN), a value between 0 and 1 that expresses the probability of diffuse emission from above the canopy when it passes through the canopy and reaches a point.

8. Maintenance

1. LAI-2050 Sensor Accessories

The lens in the probe should be wiped with a cloth moistened with evaporated water or a clean liquid. Be careful not to scratch the protective casing on the outside of the lens. Avoid wiping the lens with paper products and do not dry wipe.

The probe does not need to be recalibrated as long as the optics in the probe remain in their original position.

2. LAL2070 console

Clean only with water and do not use any other solvents.

3. Battery

With the 6D battery, the low battery symbol (L.) will appear when only 15% of the battery is left.

As long as the instrument is powered off for no more than 5 minutes, no data will be lost when replacing the battery. The battery can be replaced by unscrewing the screws on the corners of the back cover, opening the cover and removing the four screws on the battery compartment. There is no need to plug in the battery connector.

Note: Users often report damage to the instrument due to battery leakage, so the battery should be checked frequently for leakage and removed when not in use for long periods of time.

4. Restart touch

If the console is "locked" and no buttons work, the first remedy is to open the back cover, unplug the battery and reconnect it after a few seconds. If this does not work, the reboot button can be used. This button is located on the upper printed circuit board and can be seen when the back cover is opened, it is located on the right side of the board, approximately below the SETUP button. The microprocessor can be restarted by tapping this button with a small screwdriver and the memory is cleaned and all data is lost.

9. Description of function settings

0 Setup

1 X Cal

2 Y Cal

These two settings are used to enter the 5 correction values when the probe is inserted into the X and/or Y ports. When these values are entered, they are also stored in the X or Y vector (see FCT03) 。These vector values are synchronised with time; when FCT01 or FCT02 is re-edited, the X or Y vector is also updated, whether or not new values are entered.

3 Vectors

There are 6 options.

- Return Back
- CompX Perform measurements
- CompY Perform measurements
- ReselX,Y Restored to factory
- EditX Edit X vector
- EdilY Edit Y vector

Before using both probes at the same time, CompX and CompY should be performed so that both probes give the same value under the same conditions. Select either one, press the Enter key and position both probes horizontally, measure the same location and press the Enter key again. The selected vector will be corrected.

ResetX,Y reverts the vector value to the correction value saved by FCTOI and 02.

Edit X and Edit Y allow viewing and editing of the X and Y vector values. The modified values do not affect the stored correction values.

4 Resolution

Two options are available: high precision and low precision. Low precision is fine, except in low light conditions. Choosing high precision will make the instrument a little slower to respond, but the difference will not be significant.

To check whether high accuracy is required, place the instrument in a stable position and observe which ring value is the lowest. When the value fluctuates and the minimum increase in the minimum ring value exceeds 1% of this value, high accuracy is required.

5 Set Clock

6 Set Dists

Set the distance vector. The probe 5 ring has 5 values. These values are present in each file. There are three options.

- Return Return

- EditView Editors - Reset(l/cos0) set to l/cos0

  The Reset option is the machine default.

7 Set Angles

Set the angle values for each ring 7, 23, 38, 53, 68, which normally do not need to be changed, and if the angle changes, also reset the DIST value

8 l,2Channels

When the BNC interface is connected to the radiation probe of the

associated with the new angle.

The order in which the AB values are read is controlled by the user by pressing the decimal point key in the LOG program, which swaps the position of the REALTIME and SUMMARY columns so that when the REALTIME column is on the top line, the next value is A. If the REALTIME column is on the bottom line, the next value is B. Pressing the LOG key ends the reading. If the REALTIME column is on the bottom line, the next value is B. Pressing the LOG key ends the fetch.

9 Set Prompts

10 Def Log Key

This function allows the user to define the LOG key, the default value of which is 14 (Log New), and to change its setting by entering the two-digit code for the desired function after the LOG key has been pressed. However, it does not change the setting of the program that will end when the LOG key is pressed during the LOG program.

11 Log New

The new data is recorded in the order and number of records set by the user in FCT12 and in FCT11. When * appears in the top row, the next data is recorded as value A. When * appears in the bottom row, the next data is recorded as value B.

12 Log Append

Similar to FCT14 except that it is a continuation of the original file and there are no more notes to prompt for problems. The data is stored in a new file and the original file remains unchanged.

13 Bad Reading

Use this function to make changes when the B value is greater than the A value. There are three options.

- Ignored: The machine chirps for about half a second when the transmittance is greater than 1.0. This set of B values is not used in the calculation but is still present in the file and this ignored chirp is sounded when the value is recorded for the second time. In the recalculation (FCT 26) the machine chirps once for each incorrect value. This method is used when the actual values of A and B are not very close to each other.
- Let A/B = l: if the transmittance is greater than 1.0, this is considered to be l.Oo This does not change the stored data and is suitable when reading B values under canopies with few or no leaves.
- Set B/A=l: As with A/B=l, the difference is that the A value is assumed to be the sub-crown value and the B value is the supra-crown value. This should only be used in the "two-sensor approach", where the X-sensor is located under the crown and the Y-sensor is located above the crown.

The data collected can be calculated in one of the ways set in the FCT16, or it can be recalculated in another way, either with A/B=l, or B/A=l, the LAI is 0.

14 Off

Shutdown.

15 **FiIes

16 Memory Status

Displays the number of files, active storage space and total storage space. If no files have been deleted then NumFiles shows the number of files

that actually exist, if files have been deleted then NumFiles does not show the number of files that actually exist.

17 Delete

To perform a file deletion operation. As an example, there are 1-10 files, if you delete files 1-5 and keep files 6-10, then the new file number is 11, if you delete files 6-10 and keep files 1-5, then the new file number is 6.

18 Renumber

Renumbering of files to prevent broken file numbers due to file deletion operations.

19 Clear Ram

Clear out all files so that the file numbering starts from 0. This operation does not affect other data such as the calibration values stored in the instrument.

20 Edit

There are four

- Return — Back
- Slrio Obs — Removing the observations from the Space , However, no further recalculations or other
. Change Rems — Edit the notes in the selected storage
- Change Dists — Same function as FCT06 to change the DIST vector When the set DIST value is found to be.

Appendix

AH literature

B fuck Chew to

-Insert the probe into the X-port

- FCT01 input correction value
- FCT04 set to high precision
- FCT05 to determine the correct date in the instrument
- FCT11 is set to ISenseX, the order in which the values are taken and the number of repetitions.
- FCT12 sets the content of the notes
- FCT16 sets how to handle when the B value is greater than the A value
- BREAK determines that each ring (XI-X5) is responsive to light
- FCTI4 starts measuring and recording.

C matching sensors **D** Gap Test

Used to quantify errors that occur when using large-view mirror covers in non-homogeneous canopies. This is where there is very dense foliage in one part of the canopy and sparse or absent foliage in another. Because the LAI is proportional to the logarithm of the void fraction, but the probe averages linearly over the void fraction for any reading, if there are both dense and sparse areas within the probe's field of view, then the B value will be an incorrect average. If dense and sparse areas are present in different B values, They will then be correctly averaged.

- Use the 45° mirror cover.
- Set the transmittance in FCT16 to 1 °
- The DIFN is measured at the place or places where the leaves are sparsest, (i.e. LAI is measured with one probe and the D1FN value is observed.) For example, in a strip crop canopy, place the probe in the middle of two rows of crops with the view parallel to the monopole, when the least number of leaves is observed. If the gap is large and there are no leaves in the line of sight of the probe, a DIFN of 1 can be observed.
- DIFN values are measured at one or more of the densest parts of the canopy.
- The DIFN values obtained twice were used to find the error values in the canopy gap difference table.
- If the error is less than 10%, then the gap in the canopy will not affect the accuracy of the data. If it is more than 10%, then the dense part of the canopy and the gap should be measured separately, using a

45° lens cover.

How much **B** value should be measured for **E**

A simple method can be used to determine the number of B values that will bring the LA1 value at the 95% confidence limit.

- Six B values are measured first to calculate LAI values, which should include the densest and thinnest parts of the canopy.
- Calculate the SEL value for LAI.
- Use the table below to determine the number of B values.

SEL	0.	010.	020.	030.	040.	050.	060.	070.	080.	090.1
B-valu		23568II13161923								

F blade size

The table below provides the minimum distance between the blade and the probe based on the blade size and zenith angle. The minimum distance between each ring and the blade is determined by first finding the column for the angle of the mirror cover used by the probe, then subtracting the number of B readings from the value of each ring in the column and multiplying by the blade width
