# Supplementary material for: Validation of leaf area index measurement system based on wireless sensor network
Source: Sci Rep. 2022 Mar 18;12:4668. doi: 10.1038/s41598-022-08373-z (PMC8933413; doi:10.1038/s41598-022-08373-z)
Supplement: Supplementary file 3 — Supplementary Information 3. [file 41598_2022_8373_MOESM3_ESM.pdf]

Licenses for collection of plant specimens

At the request of the team of Professor Li Xiuhong of Beijing Normal University to collect plant samples. Considering the foundation, significance and contribution of the team's research, after discussion by the management of the test base, the application is now approved.

Applicant: Xiuhong Li

Approver: Junhua Bai

Approval Unit: Huailai experimental station

Time: 20 April 2015
