# Supplementary material for: Validation of leaf area index measurement system based on wireless sensor network
Source: Sci Rep. 2022 Mar 18;12:4668. doi: 10.1038/s41598-022-08373-z (PMC8933413; doi:10.1038/s41598-022-08373-z)
Supplement: Supplementary file 4 — Supplementary Information 4. [file 41598_2022_8373_MOESM4_ESM.pdf]

Statement on Experimental Research

The experiment in this article was carried out at the Huailai Remote Sensing Comprehensive Test Site of the Institute of Remote Sensing and Digital Earth, Chinese Academy of Sciences. The test site is located in the middle of the Huaiyan Basin at the junction of Hebei Province and Beijing. It is a part of the Knowledge Innovation Project of the Field Station System of the Chinese Academy of Sciences and the experimental base of the State Key Laboratory of Remote Sensing Science jointly established by the Chinese Academy of Sciences and Beijing Normal University. The State Key Laboratory of Remote Sensing Science is one of the project sponsors of this article and the work unit of the co-authors of this article. The scientific research of the station is positioned in three aspects, one is a new remote sensing earth observation theory and observation system test base, the second is a quantitative remote sensing forward and inversion model development and verification base, and the third is a quantitative remote sensing data product verification base. In order to carry out the experiment, different crops were cultivated in the field according to the needs of the experiment for use during the experiment. The experiments involved in this article need to comply with the relevant regulations of the Huailai test site, such as the "Regulations on the Management of the Public Technical Service Center of the Institute of Remote Sensing of the Earth" and so on (the relevant rules and regulations http://www.radi.cas.cn/platform/gzzd/). This article is operated and completed under the above-mentioned rules and regulations, and hereby declares.

Declarant: Xiuhong Li
Time: 14 December 2021
